# Phenotype to genotype: A new and rapid approach using whole-genome sequencing

**DOI:** 10.1371/journal.pgen.1011702

**Published:** 2025-07-14

**Authors:** McKenna Feltes, Aleksey V. Zimin, Sofia Angel, Nainika Pansari, Monica R. Hensley, Jennifer L. Anderson, Meng-Chieh Shen, Mackenzie Klemek, Yi Shen, Vighnesh S. Ginde, Hannah Kozan, Nhan V. Le, Vivian P. Truong, Meredith H. Wilson, Steven L. Salzberg, Steven A. Farber

**Affiliations:** 1 Department of Biology, Johns Hopkins University, Baltimore, Maryland, United States of America; 2 Department of Embryology, Carnegie Science, Baltimore, Maryland, United States of America; 3 Department of Biomedical Engineering, Johns Hopkins University, Baltimore, Maryland, United States of America; 4 Departments of Computer Science and Biostatistics, Johns Hopkins University, Baltimore, Maryland, United States of America; University of Pennsylvania School of Medicine, UNITED STATES OF AMERICA

## Abstract

Forward genetic screening is a powerful approach to assign functions to genes and can be used to elucidate the many genes whose functions remain unknown. A key step in forward genetic screening is mapping: identification of the gene causing the phenotype. Existing mapping methods use a bioinformatic mapping-by-sequencing approach based on allelic frequency calculations that often identify large genomic regions which contain an intractable number of candidate genes for testing. Here, we describe WheresWalker, a modern mapping-by-sequencing algorithm that identifies a mutation-containing interval and then supports positional cloning to shrink the interval, which drastically reduces the number of potential candidates, allowing for extremely rapid mutation identification. We validated this method using mutants from a forward genetic mutagenesis screen in zebrafish for modifiers of ApoB-lipoprotein metabolism. WheresWalker correctly mapped and identified novel zebrafish mutations in *mttp*, *apobb.1*, and *mia2* genes, as well as a previously published mutation in maize. Further, we used WheresWalker to identify a previously unappreciated ApoB-lipoprotein metabolism-modifying locus, *slc3a2a*.

## Author summary

Forward genetic mutagenesis screening is an unbiased approach for the identification of mutations linked to a phenotype of interest. While this approach can be a powerful tool for uncovering novel gene functions, identification of the specific phenotype-causing mutation can be time-consuming, and is a limiting factor. Here, we introduce WheresWalker, a mapping tool that uses whole genome sequencing data to identify a region of the genome linked to the phenotype. WheresWalker retrieves genetic markers from the linked interval that are used to narrow the region, until a small number of candidate genes remain; these candidates are tested using high-efficiency CRISPR/Cas9 editing. WheresWalker works in multiple species, and was applied to identify mutations from a recent forward genetic screen in zebrafish for genes that modulate ApoB-lipoprotein metabolism, a key factor in the progression of metabolic disease. Using WheresWalker, we have identified a novel locus, *slc3a2a*, that is involved in ApoB-lipoprotein biogenesis.

## Introduction

Forward genetic screening is an established and effective technique for assigning new functions to genes, and is a particularly exciting strategy to address the ~ 20% of human genes with unknown functions [1] through screens in model organisms such as zebrafish. Chemically (e.g., N-ethyl-N-nitrosourea [ENU], Ethyl methanesulfonate [EMS]) induced point mutants are powerful tools for the dissection of gene function in a variety of model organisms, but relative to more-easily identifiable genome modifications (e.g., transposon-mediated insertions, CRISPR/Cas9-mediated deletions), identifying the causative single base pair substitution that underlies a particular phenotype is significantly more challenging. For the foundational chemical mutagenesis screens of the 1980-90s, mutation identification was largely achieved through a labor-intensive positional cloning approach. Specifically, mutants were outcrossed into a polymorphic background, then successively genotyped for established polymorphic markers to identify a minimal region of the genome with perfect linkage to the phenotype (recombinant mapping); that region was then amplified and sequenced [2]. Going from phenotype to mutation identification was a process that could take years. As genomic sequencing became more efficient and affordable, mapping-by-sequencing approaches emerged which utilized transcriptomic [3], exomic [4], or genomic [5–14] datasets in order to bioinformatically identify mutations linked to the observed phenotype.

Mapping-by-sequencing has drastically improved mutation mapping. Unfortunately, many of the original mapping tools are no longer maintained. Functional, modern mapping-by-sequencing algorithms [15–17] identify the most promising mutations using “allelic frequency”, a metric calculated using read counts to determine the fraction of mutant allele at a given genomic location. When total read counts are low, for instance in repetitive or GC-rich regions or when low coverage sequencing is collected, allelic frequency is unlikely to represent the true allelic ratio. In addition, the size of the interval generated by mapping-by-sequencing is dependent on the number of animals observed and the depth of sequencing [18] which is still limiting. Coverage can be selectively increased by using RNA or exome sequencing. However, for both techniques, mutations in non-coding regions are missed, and for RNA-seq, mutations in genes that are not expressed cannot be identified. In addition, non-coding regions contain polymorphisms that can be exploited for traditional recombinant mapping, the resolution of which is only limited by the number of individuals that can be generated and genotyped.

We sought to develop a new pipeline that consists of 1) whole genome sequencing (WGS) of phenotypically mutant and wild-type pooled genomic DNA for variant detection using state-of-the-art alignment and SNP calling strategies; 2) identification of a genomic interval linked to the phenotype with a new and simple-to-use mapping-by-sequencing algorithm based on locating low-heterozygosity regions; 3) automated mapping marker identification for optional interval refinement using traditional positional cloning; and 4) candidate gene testing using efficient F0 CRISPR/cas9 protocols [19]. We have applied this highly efficient mapping pipeline, called WheresWalker, to map ENU mutants with defects in apolipoprotein-B (B-lp) biosynthesis that we recently generated in a forward genetic screen in zebrafish. WheresWalker identified the correct locus for novel zebrafish alleles of *mttp*, *apobb.1*, and *mia2*, validating the strategy. To demonstrate the utility of WheresWalker beyond zebrafish, we show that the pipeline also successfully identifies a previously described mutation in maize. Finally, in a matter of weeks, we used WheresWalker to map a novel dark yolk mutant, *zion*, to *slc3a2a*, and show that this locus is linked to B-lp synthesis. Thus, we present WheresWalker as a powerful new tool for allele discovery.

## Description of the method

### Bulk segregant analysis

For recessive mutations, the causative locus and surrounding genomic region will be homozygous in mutant animals, while regions outside of the locus will be more heterozygous as zebrafish are highly polymorphic [20]. To leverage this principle we designed an algorithm that utilizes whole genome sequencing (WGS) data from mutant and wild-type sibling genomic DNA to identify regions of the genome that are more homozygous in mutant animals. Heterozygous adults are incrossed to generate clutches which are sorted by phenotype; 20–30 larvae per phenotype are pooled and used to generate phenotypically wild-type (+/+ and +/-) and phenotypically mutant (-/-) genomic DNA for WGS (Fig 1.1). Crude genomic DNA from individual mutant (-/-) larvae is prepared in parallel for downstream fine-mapping. WGS data is aligned and evaluated for points of variance using POLCA [21], a fast and accurate genome polishing tool that generates a report on genome variance in the form of variant call format (VCF) files (Fig 1.1).

**Fig 1:**
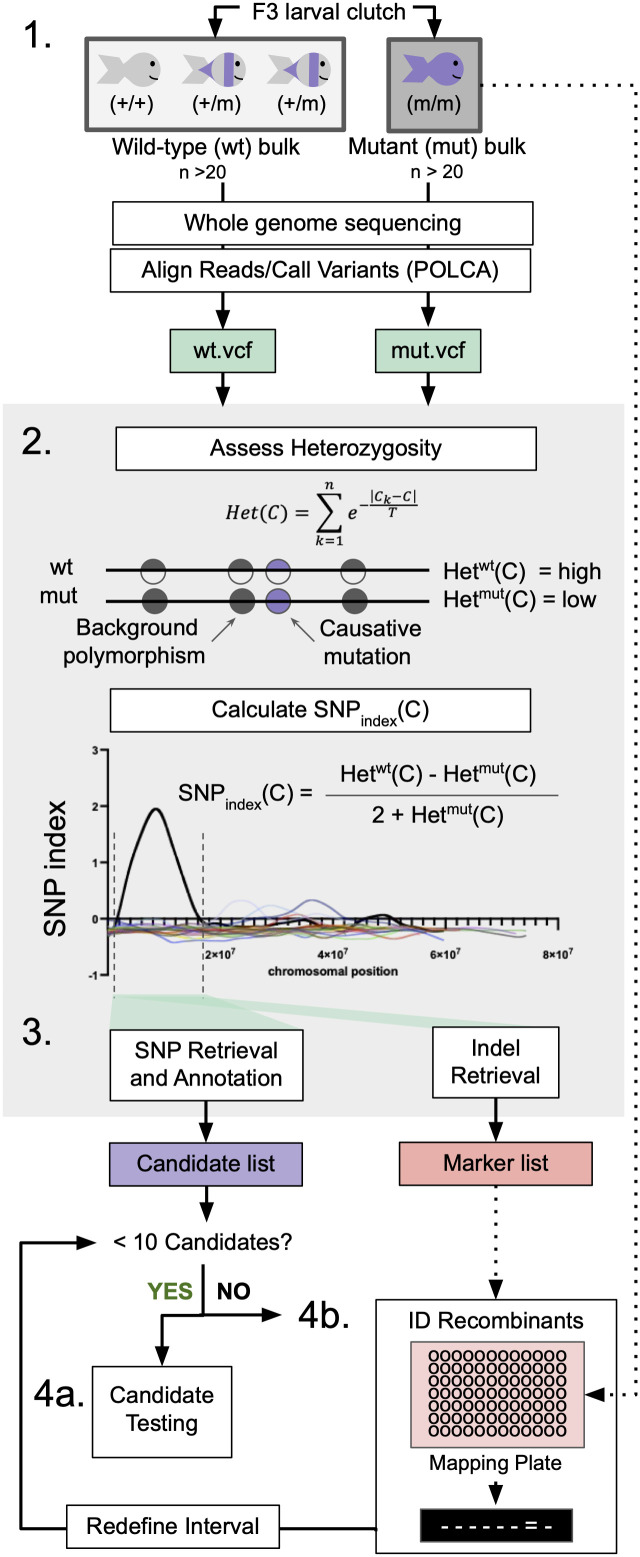
WheresWalker pipeline utilizes WGS data to identify segregating SNPs and indels. 1. Bulk Segregant Analysis: animals are sorted by phenotype and pooled to generate wild-type (wt) and mutant (mut) genomic DNA for whole-genome sequencing. Sequencing data is aligned and evaluated for variance using POLCA which outputs VCF files for wt and mut samples. gDNA for additional mutant animals can be saved for downstream analyses. 2. Heterozygosity is calculated in a sliding window across wt and mut genomes, where C is the coordinate at the center of each 10,000 bp window. These values are used to calculate the SNP index in order to define a homozygous interval; dashed lines indicate interval bounds. 3. Whereswalker extracts SNPs and indels that segregate appropriately with the mutant phenotype to generate a list of candidate SNPs and a list of indel markers. Steps 2 and 3 are executed in a single command by the WheresWalker script. 4a. If sufficiently few candidates have been identified, the genes can be targeted with CRISPR/Cas9. 4b. If the number of targets is intractable, the interval can be refined by identifying recombinants. This can be repeated until a sufficiently short candidate list has been generated.

### Homozygosity mapping

To identify regions of homozygosity, VCF files for wild-type and mutant samples as well as the appropriate genome assembly file are supplied and the WheresWalker script is executed. The WheresWalker algorithm uses 10 Kbp sliding windows to quantify the mean degree of heterozygosity for mutant Het^mut^(C) and wild-type Het^wt^(C) datasets by counting the number of heterozygous points in each window, where *C* is the coordinate of the window center (Fig 1.2). Relative homozygosity, or SNP index is calculated as follows: *SNP*_*index*_*(C)=*(Het^wt^(C)*-*Het^mut^(C)/(2 + Het^mut^(C)*).* In regions where mutants are less heterozygous (more homozygous) than wild-type siblings, Het^wt^(C) will be much larger than Het^mut^(C), causing the *SNP*_*index*_*(C)* to be high and positive. To smooth out noise, we apply a moving average filter (MA) to the *SNP*_*index*_ using a window width of 750 kb (an adjustable parameter; this value was chosen because it represents 1 cM for zebrafish). The genome is then scanned for the highest value of *MA(SNP*_*index*_*(C)),* and an interval around the maximum, bounded on each side by *MA(SNP*_*index*_*(C))=*0, is selected for further analysis (Fig 1.2).

### SNP retrieval

For all of the variants inside the defined homozygous interval, WheresWalker adds gene-based annotation using a modified version of ANNOVAR [22]. In addition, WheresWalker filters the variants to generate a report on the most promising mutations based on 1) appropriate segregation with the mutant phenotype, 2) likelihood to have resulted from ENU mutagenesis, and 3) potential to disrupt gene function (Fig 1.3). To select only appropriately segregating variants, WheresWalker considers the number of reads that agree with the reference genome sequence (RO) and the number that do not (AO). A variant must be sufficiently mutant (RO < 2 and AO ≥ 2) in the mutant sample and either heterozygous (1 > RO/AO < 4) or not observed in the wild-type sample. To identify mutations that are likely to be the result of ENU mutagenesis, only single and double base pair variants are selected. Finally, to select the variants with non-synonymous mutations, start codon loss, and stop codon loss/gain are reported. Optionally, if provided, WheresWalker adds SIFT values, which are available for previously identified variants and estimate the degree to which a known variant may be detrimental [23]. This feature allows for previously undocumented variants (likely ENU-induced), or documented variants with predicted detrimental consequences (likely non-ENU, but still potentially causative) to be prioritized. WheresWalker ultimately outputs both an unfiltered and filtered variant list.

### Positional cloning module for fine mapping

If the resulting filtered variant list represents a sufficiently small number of gene candidates, the genes can be targeted by CRISPR/Cas9 and F0 animals can be monitored for phenotype development (Fig 1.4a). However, this may not be feasible or practical when numerous promising candidates have been identified. To further narrow the number of possible candidates, WheresWalker automatically generates a list of potential mapping markers by identifying insertions and deletions (indels) in the homozygous interval that appropriately segregate with the mutant phenotype. This list can be used to design primers for selected indel markers for genotyping of individual mutant (-/-) larvae to identify recombinant animals (Fig 1.4b). Recombination frequency (Rf = recombinants/total) is calculated, and the distance to the causative locus is estimated (Rf*cM, zebrafish cM = 0.74 Mb [24]). Candidate genes within the bounds of the observed recombination points, and nearest the estimated locus position can then be prioritized and tested using an F0 CRISPR approach.

### Executing WheresWalker

WheresWalker is available for download at https://github.com/alekseyzimin/WheresWalker. WheresWalker requires mutant and wild-type sibling vcf files, a genome assembly file, and an optional SIFT file. WheresWalker is executed with a single command and generates output files in the plain text format for downstream analysis, which we performed using R/RStudio.

## Verification and comparison

### Forward genetic screen uncovers 28 dark yolk mutants

ApoB-containing lipoproteins (B-lps) are essential for transporting lipid between tissues, but in excess they play a causative role in a collection of metabolic disorders that impact over 1.2 billion people worldwide [25]. B-lps are synthesized in the liver and intestine in the ER lumen where microsomal triglyceride transport protein (MTP) loads lipid cargo onto Apolipoprotein B (ApoB) to form a lipid-filled particle. The ER transmembrane protein, TALI, mediates export of B-lps from the ER [26]. Like humans, zebrafish synthesize B-lps in the liver and intestine. In addition, in larval stages, B-lps are also synthesized in the area surrounding the yolk, the yolk syncytial layer, from maternally deposited yolk lipid. As is observed in zebrafish mutants of *apobb.1* (ApoB) [27], *mttp* (MTP) [28], and *mia2* (TALI) [29], disruption of B-lp synthesis results in abnormal lipid accumulation in the yolk syncytial layer which increases the opacity of the tissue. This “dark yolk” phenotype can be observed using low powered light microscopy with transmitted light.

**Fig 2:**
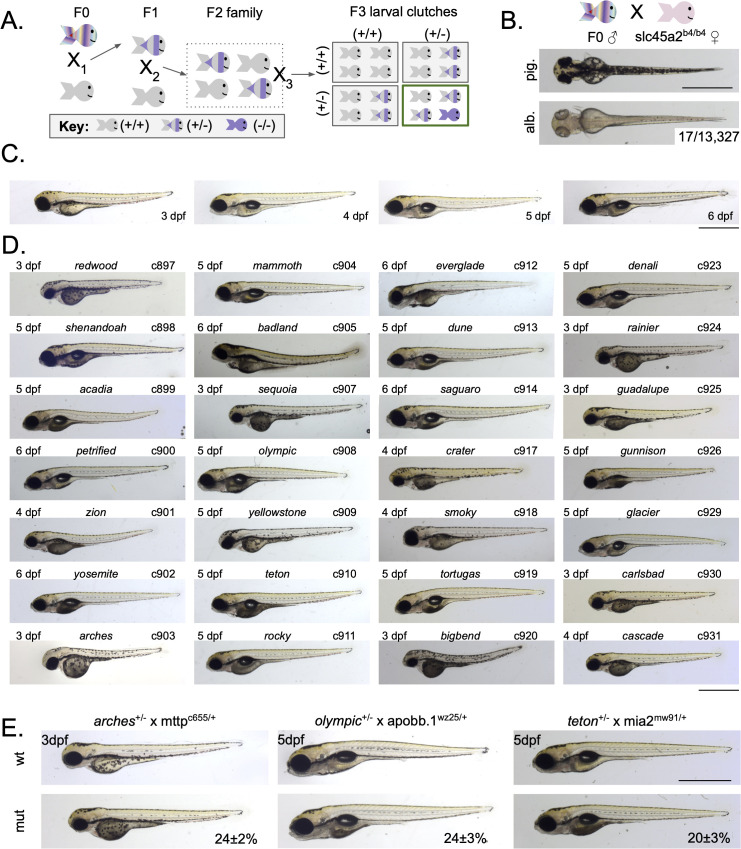
Forward genetic screen identifies 28 dark yolk mutants. A) Generation of mutant families using a standard forward genetic F3 screening scheme. B) Single locus hit rate for the *slc45a2* locus was determined by crossing male founders to *slc45a2*^*b4/b4*^ females and screening for albinism in the offspring; representative pigmented (pig.) and albino (alb.) 3 days post-fertilization (dpf) larvae are shown. C) Representative images of wild-type (wt) zebrafish from 3-6 dpf. D) Representative images of identified mutants; age, mutant name, and allele ID (cXXX) are noted. E) Screen mutants were crossed to known dark yolk mutants so that progeny could be evaluated for dark yolk. Representative images for 3 mutants that fail to complement known dark yolk loci. Phenotype frequency is reported as mean ± standard deviation. For *arches*: N = 4 clutches, n = 375 animals; For *olympic*: N = 5 clutches, n = 443 animals; For *teton*: N = 4 clutches, n = 396 animals. For all panels, scale bar represents 1 mm.

In an effort to identify new modifiers of B-lp biology, we initiated a traditional F2 forward genetic screen looking for mutant families exhibiting the dark yolk phenotype. A founding (F0) generation of adult zebrafish males were exposed to ENU to introduce point mutations into the germ cells (Fig 2A). A single locus hit frequency was measured at the albino locus (*slc45a2*) to be 0.13% which is comparable to previous ENU screens in zebrafish (Fig 2B) [4,30]. F0 founders were outcrossed to wild-type females to generate an F1 generation (Fig 2A, X_1_); individual F1 fish were outcrossed to Fus(*ApoBb.1-nanoluciferase*)^+/-^ to generate F2 families in a background that allows for quantification of B-lp [31] (Fig 2A, X_2_). Blind incrosses of F2 families were performed to generate F3 larvae which were screened for yolk phenotypes (Fig 2A, X_3_). For each F2 family, an average of 6 mating pairs were evaluated, giving a ~ 0.82 (1-0.75^6^) probability of pairing two heterozygous F2 fish in at least one of the blind incrosses. This probability (1-0.75^n^) was used to calculate the fraction of the genome screened for each of the 1,023 families and summed to equal 814 genomes which represents 1.05X genomic coverage. Twenty-eight dark yolk mutants were identified (Fig 2D), of which 27 produced the phenotype in Mendelian ratios and could be recovered in subsequent generations (S1A Fig).

Alleles of *mttp* [28], *apobb.1* [27], *mia2* [29], *dgat2* [32] and *pla2g12b* [33] have already been reported to produce the dark yolk phenotype. In order to determine if these loci were represented in our mutant collection, each novel mutant was crossed to the known dark yolk alleles to generate progeny for evaluation. Using this complementation approach, 4 *mttp* alleles (*arches*, *bigbend*, *guadalupe*, *carlsbad*), 2 *apobb.1* alleles (*mammoth*, *olympic*), 2 *mia2* alleles (*teton*, *dune*), and 2 *dgat2* alleles (*rainier*, *cascade*) were identified (Figs 2E and S2B–S2E). The other 17 mutants represent at least 13 unique loci, though complementation testing is still ongoing. *pla2g12b* was not identified in this screen, suggesting saturation was not reached. Three of these known dark yolk mutants (*mttp^arches^*, *apobb.1^olympic^*, *mia2^teton^*) were selected to test the WheresWalker mapping pipeline.

### WheresWalker identifies correct chromosome for three known dark yolk loci

**Fig 3:**
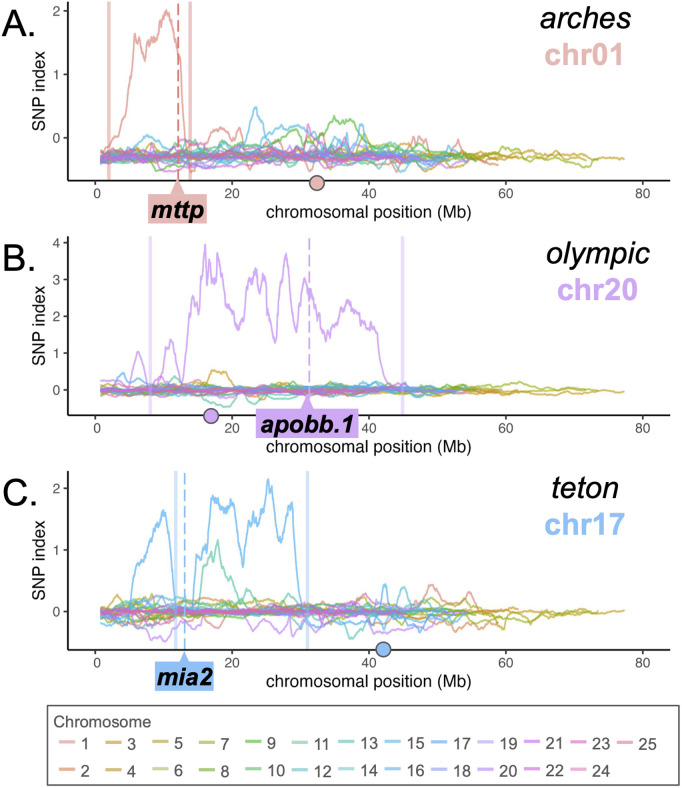
WheresWalker identifies the correct chromosomal region for three dark yolk loci. Profile of SNP index across all chromosomes for *arches* (A) *olympic* (B) and *teton* (C) mutants. Solid lines indicate left and right bounds of the interval selected by WheresWalker, dashed lines indicate the position of the causative locus. Circles on the x axis indicate the approximate position of the centromere.

WGS data was collected from wild-type and mutant genomic DNA generated from an incross of F2 *mttp*^*arches/*+^, *apobb.1*^*olympic/*+^, or *mia2*^*teton/*+^ parents. 20–30 progeny (see Table 1) were pooled for each phenotype to generate genomic DNA for sequencing. For each set, VCF files generated by POLCA were submitted to WheresWalker. For each of the 3 mutants, visual assessment of the SNP index for every chromosome indicated a single chromosome with elevated SNP index (Fig 3A–3C). For *arches*, an interval of 11.40 Mb on chromosome 1 was selected which contained the causative locus, *mttp*. A 36.86 Mb interval on chromosome 20 containing *apobb.1* was selected for *olympic* (Fig 3B), and a 17.56 Mb interval on chromosome 17 containing *mia2* was selected for *teton* (Fig 3C). In all cases, the pipeline was sensitive enough to select the correct chromosome and the genomic interval of the causative locus even without an outcross.

**Table 1:** Summary of WGS datasets.

Mutant	Background	Bulk	Coverage	Chromosome	Interval (Mb)	Interval size (Mb)	Mean local SNP index	Indels/Mb	SNPs/Mb	Candidate genes	Candidate SNPs	nonsense	non-synonymous	startloss	stoploss	splicing
**mttp:*arches***	AB	4x5	30X	1	1.99 - 13.87	11.88	1.24	327	230	62	127	3	124	0	0	0
	WIK	4x5	30X	1	5.25 - 28.08	22.83	4.15	553	105	47	100	1	98	1	0	0
	WIK	1x28	30X	1	4.89 - 46.08	41.19	2.19	253	188	143	328	7	319	0	1	1
	WIK	3x10	30X	1	5.1 - 28.17	23.07	3.31	493	194	81	191	3	187	1	0	0
	WIK	NA	30X	1	5.22 - 28.15	22.93	5.30	521	133	64	133	2	130	1	0	0
	WIK	3x10	60X	1	4.98 - 25.76	20.78	2.95	385	174	71	182	3	178	1	0	0
	WIK	3x10	15X	1	5.24 - 28.24	23	3.42	609	229	105	231	3	228	0	0	0
	WIK	3x10	5X	1	5.27 - 27.42	22.15	0.77	311	262	110	229	3	225	1	0	0
**apobb.1:*olympic***	AB	3x10	30X	20	8.03 - 44.89	36.86	2.66	506	85	102	235	2	230	3	0	0
	WIK	3x10	30X	20	0.76 - 55.17	54.41	2.96	760	124	156	388	10	375	2	1	0
	WIK	3x10	15X	20	0.76 - 55.16	54.4	2.45	681	127	165	394	11	381	0	2	0
	WIK	3x10	5X	20	0.76 - 55.14	54.38	0.54	313	199	266	563	18	540	1	4	0
**mia2*:teton***	AB	2x15	30X	17	11.76 - 31.01	19.25	0.04	313	118	57	101	1	99	0	1	0
	WIK	2x15	30X	17	0.21 - 30.93	30.72	3.88	441	77	43	103	3	100	0	0	0
	WIK	2x15	15X	17	1.62 - 31.21	29.59	3.53	503	104	68	140	1	139	0	0	0
	WIK	2x15	5X	17	9.37 - 31.21	21.84	0.81	320	204	97	192	4	188	0	0	0

### Background heterogeneity improves SNP index

**Fig 4:**
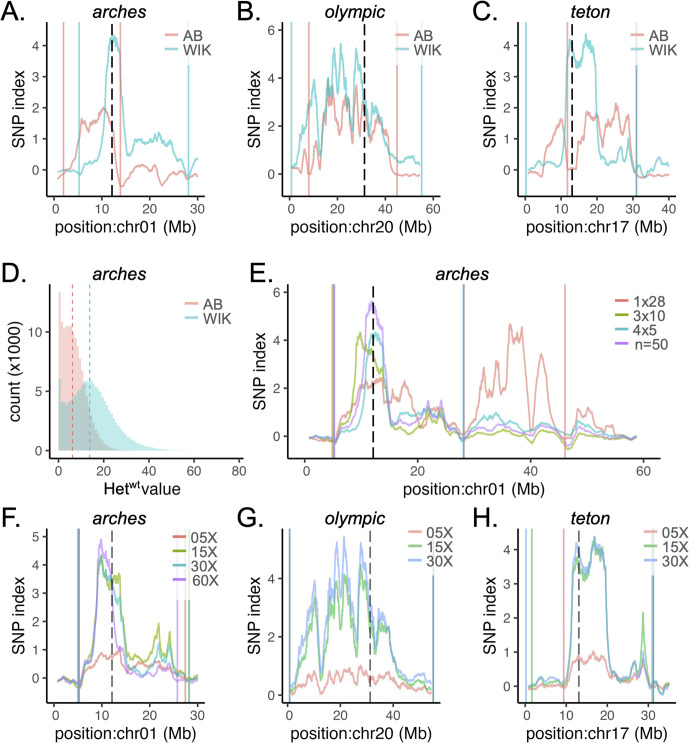
Background heterogeneity and sequencing depth improve WheresWalker SNPindex. A-C) regional SNP index profile for datasets generated in an AB or WIK background for *arches* (A), *olympic* (B), and *teton* (C) mutants. D) Het^wt^ distribution for *arches* AB 4x5 and WIK 4x5 datasets. Binwidth is 1. Dashed lines mark the median (AB: 6.163692, WIK: 13.81362). E) Regional SNP index for *arches* datasets generated from different combinations of clutches: 1x28, 3x10, or 4x5 (clutch x animals per clutch). The n = 50 dataset was generated by combining 15X coverage datasets from the 3x10 and 4x5 datasets to generate a 30X dataset representing 50 animals from 4 clutches. F-H) Regional SNP index profile for the *arches* WIK, 3x10 (F), *olympic* WIK (G), and *teton* WIK (H) datasets with sequencing coverage simulated at 05X, 15X, or 30X. For *arches* WIK, 3x10, additional reads were collected from the original sample to generate an ~ 60X dataset. For SNP index plots, solid lines indicate left and right bounds of the interval selected by WheresWalker, dashed lines indicate the position of the causative locus.

Conventional mapping relies on an outcross to a different wild-type strain to introduce microsatellite markers for recombinant mapping. We hypothesized that the introduction of polymorphisms in this way would also improve bioinformatic interval picking. To test this, *mttp*^*arches*^, *apobb.1*^*olympic*^, and *mia2*^*teton*^ F2 (*arches*, *olympic*) or F3 (*teton*) heterozygous animals were outcrossed to the WIK wild-type strain to generate *mttp*^*arches/*+^, *apobb.1*^*olympic/*+^ and *mia2*^*teton/*+^ in the WIK background. These fish were incrossed to generate mutant and wild-type progeny which were pooled and sequenced to generate VCF files for WheresWalker. As in the original datasets, the interval selected contained the causative locus for each mutant (Table 1, S2A, S2H and S2K Fig). Relative to intervals selected in the AB background, intervals in the WIK background were wider, however, the causative locus was more likely to be close to the center of a major SNP index peak and the mean SNP index value in the 2 cM surrounding the mutation was higher (Table 1, Fig 4A–4C). We observe a shift toward larger Het^wt^(C) values in the WIK background (median Het^wt^(C) = 13.81362) relative to the AB background (median Het^wt^(C) = 6.163692) for the *arches* wild-type samples (Fig 4D), confirming heterogeneity increased with outcross.

We predicted that increasing the genetic diversity represented in the bulk samples by increasing the number of clutches represented in each bulk would further improve peak quality. To test this, samples were generated from *mttp*^*arches*^ which represented 1 clutch (n = 28, 1x28) or 3 clutches (n = 30, 3x10) in the WIK background and compared them to the original WIK bulk which was made up of fewer animals but represented 4 clutches (n = 20, 4x5). In addition, we combined 15X coverage WIK 4x5 and 3x10 datasets to generate a 30X coverage dataset representing 50 animals from 4 unique clutches (n = 50). For each set, an interval containing *mttp* on chromosome 1 was selected (Table 1, S2A–S2D Fig), but the selected interval was almost 2X larger when only one clutch was represented. Datasets representing 3 + clutches had decreased interval size and increased mean SNP index around the mutation (Fig 4E–4G, Table 1). Heterogeneity was markedly increased in the dataset representing 50 animals (1x28: 12.80365, 3x10: 13.5894, 4x5: 13.81362, n = 50: 28.94921, S3 Fig) and generated the highest SNP index for the *mttp* locus, but did not drastically narrow the interval size (Table 1). These data suggest that increasing the number of individuals represented improves SNP index (4x5 vs. n = 50), and increasing the number of clutches represented dramatically narrowed interval width (1x28 vs. 3x10). It is important to note that only one single-clutch bulk was analyzed, leaving the possibility that, by chance, this single clutch had particularly low polymorphism leading to the selection of a larger interval. Using multiple clutches to generate bulks decreases the chance that the sample is not polymorphic enough for mapping.

### Increased sequencing depth decreases the number of SNP candidates

To test genomic coverage requirements, existing datasets were trimmed to represent 5X and 15X coverage for the *arches* WIK 3x10 dataset. To generate a 60X dataset, an additional 30X coverage was collected for the *arches* WIK 3x10 and combined with the original 30X set. Increasing coverage above 5X did not significantly affect the size and location of the interval selected but substantially increased the SNP index near the mutation (Table 1, Figs 4F and S2F–S2G). Increasing coverage from 15X to 30X did not change the qualitative appearance of the interval (Fig 4F), but did reduce the number of segregating SNPs (Table 1). A similar effect was observed at *apobb.1* and *mia2* loci (Table 1, Figs 4G–4H and S2H–S2M). Increasing coverage from 30X to 60X by adding more reads led to a slightly smaller interval, but SNP index was lower at the *mttp* locus. The number of candidate genes in each interval was determined by selecting only genes with splicing or exonic missense or nonsense mutations (Table 1). On average, increasing coverage from 5X to 15X reduced the number of candidate genes by 24 ± 17% and increasing coverage from 15X to 30X reduced the number of candidate genes by an additional 22 ± 16%. For the *arches* WIK 3x10 dataset, increasing coverage to 60X reduced the number of candidates by 12%.

### WheresWalker maps previously described maize mutant, *vns*

We next applied WheresWalker to a publicly available WGS dataset for a mutation in maize (*Zea mays*) called *very narrow sheath* (*vns*) which was mapped to the *defective kernel 1* (*dek1*) locus [34]. The original authors attempted to identify the causative locus using the “MutMap” strategy, where homozygous positions in a pooled mutant sample are mapped, but were unable to identify a single peak until unwanted SNPs were manually filtered out using sibling and parental sequencing datasets plus HapMap and SNP databases. Similarly, in our own analysis, we find that even with VCF files generated from a more recent genome assembly (ZM-b73 5.0), plotting homozygous SNPs in the mutant sample identifies two chromosomes of interest: NC_050096.1 and NC_050104.1 (S4A Fig). To test if WheresWalker could correctly map the *vns* mutation, VCF files were generated from the available WGS data for *vns* mutant and sibling pools which were inputted to WheresWalker. Compared to the zebrafish datasets we collected, the maize data represents a smaller number of individuals (9 for each phenotype), and had a larger proportion of low heterozygosity windows (S4C Fig). However, even with relatively low heterogeneity and moderate sequencing coverage (17X), WheresWalker was able to identify a 75.71 Mb interval on chromosome 1 containing *dek1* (S4B Fig). The *dek1* locus was ~ 3 Mb from the highest SNP index peak. The WheresWalker output was comparable to the manually filtered homozygous SNP profile that led to the original mapping to the *dek1* locus [34], but was completed in a single, automated step and did not require a parental sequence. These data demonstrate the utility of WheresWalker over existing methods and beyond zebrafish.

### Bulk Segregant Analaysis homozygosity mapping mirrors SNPindex

The WheresWalker algorithm quantifies homozygosity by identifying regions that are less heterozygous in the mutant dataset compared to wild-type siblings. A wild-type sibling dataset is an ideal sample for comparison because the siblings possess the same background polymorphisms but are heterozygous at the causative mutation. To test if a more direct measure of relative homozygosity was as effective as the WheresWalker approach, we developed a simple script to identify SNPs that were homozygous in the mutant sample and heterozygous in the sibling sample. We used this script to extract homozygous points in *mttp*^*arches*^, *apobb.1*^*olympic*^, and *mia2*^*teton*^ datasets and plotted the homozygous SNP density to compare with the WheresWalker output. Only ~40–2000 homozygous points were identified/dataset. However, for each dataset, homozygous SNPs concentrated on the correct chromosome (S5A Fig) and were distributed similarly to the WheresWalker SNP index (S5B and S5C Fig).

### Recombinant mapping narrows the region of interest to identify a nonsense allele of *mttp*

**Fig 5:**
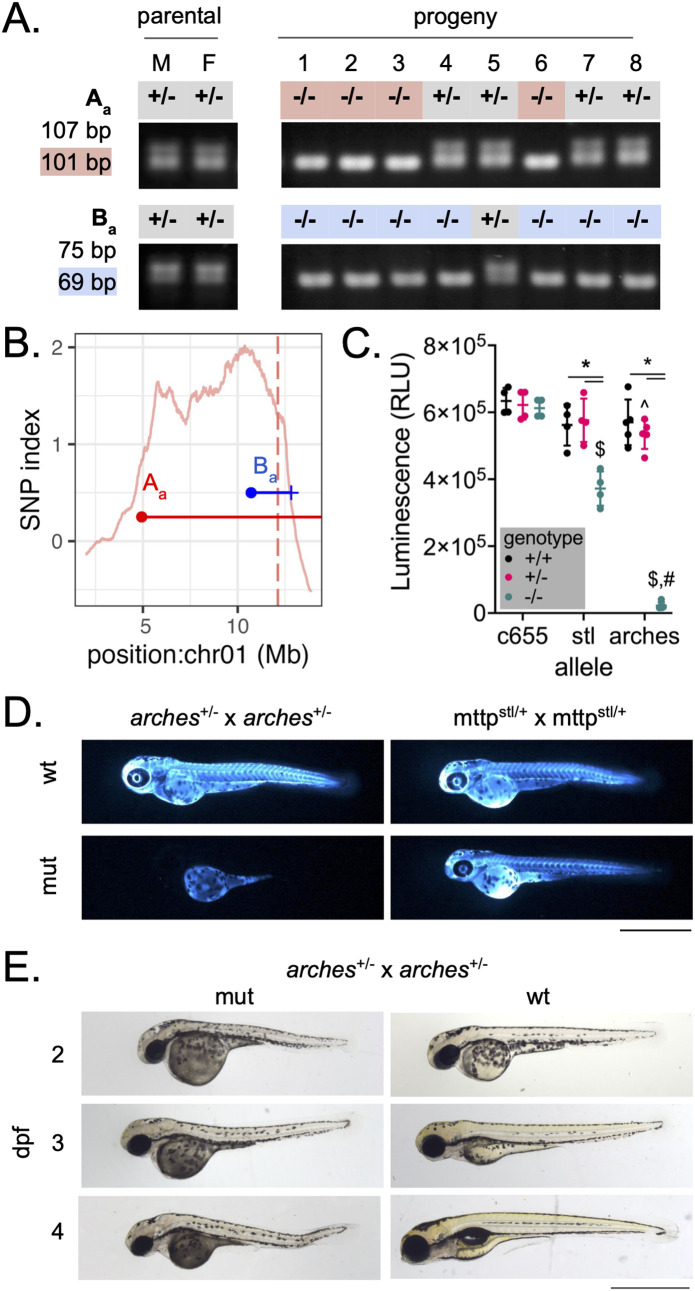
Recombinant mapping narrows region of interest to identify a loss-of-function allele of *mttp.* A) markers A_a_ and B_a_ were outputted by WheresWalker and used to genotype *arches* mutants in order to identify recombinants. M and F denote male and female parents, respectively. PCR product sizes for wild-type and mutant (highlighted) products are as indicated. B) Points representing A_a_ (red) and B_a_ (blue) marker locations and horizontal lines representing the estimated distance to the causative mutation are overlaid on the SNP index for the *arches* interval (chromosome 1: 2.46-13.86 Mb). The vertical dashed line indicates the location of *mttp*, the causative locus. C) quantification of ApoBb.1-nanoluciferase levels at 3 dpf. Mean ± standard deviation, N = 4-5 clutches, n = 2-8 animals/datapoint. P < 0.05 by two-way ANOVA with Geisser-Greenhouse correction and Tukey’s multiple comparisons test. * vs. respective wild-type, ^ vs *c655*/ + , $ vs *c655*/c655, # vs *stl*/stl. D) Images of whole-animal ApoBb.1-nanoluciferase distribution in *arches* and *stl* mutants at 3 dpf. E) Brightfield images of mutant and wild-type animals from 2-4 dpf. For D and E, scale bar represents 1 mm.

With 30X genomic coverage, WheresWalker picks an interval of ~10–50 Mb which represents 0.5-3% of the zebrafish genome. This is a substantial reduction in the total amount of genomic space to search for the causative SNP but is still quite large and contains hundreds of candidate genes (Table 1). Whereswalker automatically extracts indels that segregate with the phenotype that can be used for recombinant mapping to narrow the mapped interval. To test this module, PCR primers were designed around mapping indel markers (“A_a_” and “B_a_”) outputted by WheresWalker on either side of the *mttp*^*arches*^ interval. *mttp*^*arches*/+^;A_a_^+/-^;B_a_^+/-^ parental fish were incrossed to generate *mttp*^*arches*^ mutant progeny which were collected and genotyped for markers A_a_ and B_a_ (Figs 5A and S6A). Most of the 35 mutant progeny were homozygous mutant at both markers (A_a_^-/-^; B_a_^-/-^), but 12/35 for marker A_a_ and 1/35 for marker B_a_ were heterozygous indicating a recombination event occurred, unlinking the marker from the phenotype. Because recombination frequency is a function of the linear distance between two genetic loci, these results indicated that marker A_a_ was farther from the mutation than marker B_a_. Further, in the sole recombinant for marker B_a_, animal 5, recombination was also observed at marker A_a_ suggesting both markers were on the left side of the mutation. These data exclude from consideration the region of 2.46 Mb (original interval bound) to 10.71 Mb (marker B_a_) as the location of the causative locus. The remaining 3.16 Mb region (10.71-13.87 Mb) contains 52 candidate SNPs in 24 unique genes, and a single nonsense mutation, which is in *mttp* (ENSDARG00000008637: ENSDART00000015251: exon17: c.C2475A: p.C825X) and was confirmed by Sanger Sequencing (S6B Fig). Further, an estimation of the distance to the causative locus, calculated using the recombination frequency, predicted markers A_a_ and B_a_ to be 25 and 2 Mb away (Fig 5B). *Mttp* was within the bounds of both markers and was just 1.4 Mb from marker B_a_. The nonsense mutations observed in the *olympic* mutant at the *apobb.1* locus (chr20:31279958 T > A, ENSDARG00000022767: ENSDART00000176187: exon22:c.T6651A: p.Y2217X) and the *teton* mutant at the *mia2* locus (chr17:13068742 C > A, ENSDARG00000099973: ENSDART00000188311: exon5: c.G403T: p.E135X) likely underlie the phenotype in these mutants.

The *mttp*^*arches*^ mutation introduces a premature stop codon in exon 17 leading to a 59 bp truncation. A similar truncation, observed in a human patient with abetalipoproteinemia, was shown to disrupt the binding of the *mttp* protein product, MTP, with PDI which is essential for function [35]. We therefore predicted that the *arches* mutants would have a severe phenotype and sought to compare them to previously studied zebrafish *mttp* alleles *c655* and *stl* which have deficiencies in triglyceride and triglyceride/phospholipid transfer to ApoB, respectively [28]. ApoB quantity was measured using the LipoGlo reporter system [31] in *mttp*^*arches*^, *mttp*^*c655*^, and *mttp*^*stl*^ mutants (Fig 5C). At 3 dpf *mttp*^*c655*^ have wild-type levels of ApoB while *mttp*^*stl*^ levels are reduced by ~50%. In contrast, ApoB was hardly detectable in *mttp*^*arches*^ embryos suggesting very few B-lp particles were produced. This finding was further confirmed when fixed whole embryos were assessed for ApoB localization: observable ApoB signal was restricted to the yolk syncytial layer, the B-lp synthetic tissue during embryonic stages [31,36–38] (Fig 5D). While *mttp*^*stl*^ and *mttp*^*c655*^ survive to adulthood [28], *mttp*^*arches*^ fish exhibit yolk retention, develop tissue necrosis early in development (Fig 5E), and do not survive past larval stages, further illustrating the severity of the novel *arches* allele. The *mttp*^*stl*^ allele was previously thought to be null, but, compared to the even more severe *mttp*^*arches*^ phenotype, it is clear that the *mttp*^*stl*^ allele does retain some important function and would be better described as hypomorphic.

## Application

### *zion* maps to *slc3a2a*, a novel dark yolk locus

**Fig 6:**
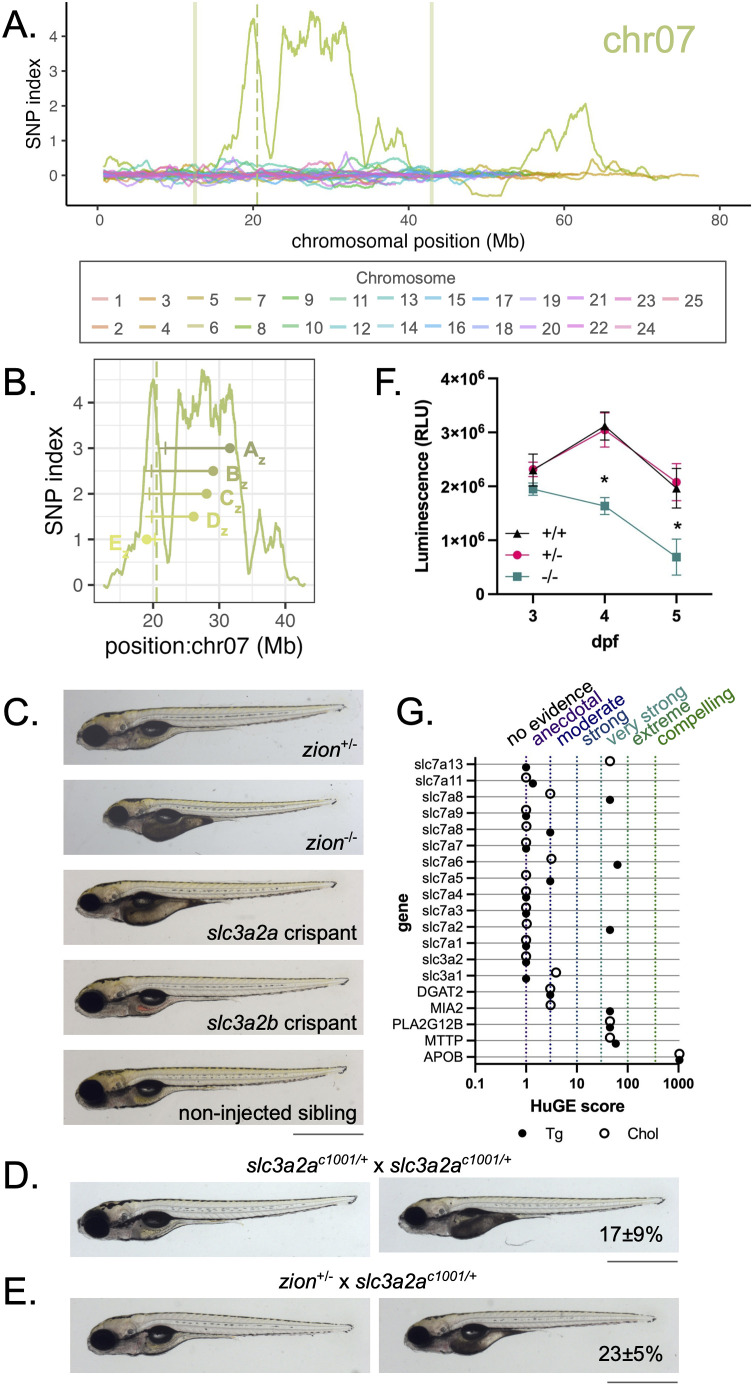
*slc3a2a*^*zion*^ is a novel regulator of B-lp metabolism. A) Elevated SNP index is observed on chromosome 7 in *zion* mutants; WheresWalker selected an interval from 19.03-26.02 Mb which was further analyzed. Solid vertical lines indicate interval bounds on chromosome 7. The vertical dashed line indicates the position of *slc3a2a* on chromosome 7. B) Mutant animals were genotyped for polymorphisms at 31609203 (A_z_), 29087847 (B_z_), 28090051 (C_z_), 26124850 (D_z_), and 19034592 (E_z_) bp to identify recombinants and predict the distance to the causative mutation. Points representing marker locations, and horizontal lines representing the estimated distance to mutation are overlaid on the SNP index for the interval. C) Representative images of larvae after editing at *slc3a2a* (dark yolk 41 ± 16%, N = 5, n = 248) and *slc3a2b* loci; non-injected larvae, as well as *zion*^+/?^ and *zion*^-/-^ siblings are shown for comparison. D) *slc3a2a*^*c1001/*+^ in-cross generates larvae with the dark yolk phenotype; dark yolk frequency is shown as mean ± standard deviation, N = 3, n = 876. E) *slc3a2a*^*c1001/*+^ crossed to *zion*^+/-^ generates larvae with the dark yolk phenotype; dark yolk frequency is shown as mean ± standard deviation, N = 3, n = 299. For panels D-F, animals are 5 dpf, scale bar represents 1 mm. F) ApoBb.1-nanoluciferase quantification in *zion* mutants and siblings. Mean ± standard deviation, N = 3, n = 2–14, outliers were removed by the ROUT method (Q = 1%). P < 0.05 by two-way ANOVA with Geisser-Greenhouse correction and Tukey’s multiple comparisons test. * vs. + /- and +/ + . G) HuGE scores for SLC3 and SLC7 genes quantify the association of human variants with serum triglyceride (Tg) and total cholesterol (Chol). Genes with established links to B-lp synthesis are shown for comparison. HuGE association categories are noted on the top.

We applied WheresWalker to map *zion*, one of the novel dark yolk mutants identified in our screen. WGS datasets were collected for wild-type and mutant samples which were inputted to POLCA [21] to generate VCF files that were submitted to WheresWalker. The pipeline selected a 30.4 Mb interval on chromosome 7 (Fig 6A, Table 2) which contained 150 exonic mutations in 80 genes. Indels, from the WheresWalker output, were selected and 5 markers (A_z_-E_z_) were optimized for recombinant mapping. A total of 121 dark yolk larvae from an incross of a single parental pair of *zion*^+/-^ adults were collected and genotyped for each marker (S7A and S7B Fig). Mapping reduced the region of interest to ~7 Mb between markers D_zion_ and E_zion_ (19.03-26.12 Mb) (Fig 6B); this region contained 42 exonic mutations in 25 genes. The recombination frequency was used to predict the distance to the causative locus from all markers, which averaged to 20.23 ± 0.96 Mb (S7C Fig). This ~2 Mb region contained mutations in 8 genes including 12 nonsynonymous and 1 nonsense SNP. The single nonsense mutation in *slc3a2a* (ENSDARG00000036427: ENSDART00000052917: exon7: c.C1012T: p.Q338X) was prioritized as the top candidate (S7A Fig). CRISPR guides targeting *slc3a2a* were injected into 1-cell zebrafish embryos with Cas9 to induce editing. Guides targeting the ohnolog of *slc3a2a*, *slc3a2b*, were also tested. At 4 dpf, *slc3a2a* injected larvae phenocopied the *zion* dark yolk phenotype, whereas no dark yolks were observed in *slc3a2b* crispants (Fig 6C). Editing at *slc3a2a* and *slc3a2b* loci was confirmed by PCR (S8B and S8C Fig).

**Table 2:** *zion* WGS summary.

Mutant	Chromosome	Interval (Mb)	Interval size (Mb)	Mean local SNP index	Indels/Mb	SNPs/Mb	Candidate genes	Candidate SNPs	nonsense	non-synonymous	startloss	stoploss	splicing
**zion**	7	12.53 - 42.96	30.43	3.52	412	123	80	150	3	146	1	0	0

To further confirm *slc3a2a* as the causative locus, CRISPR was used to generate an *slc3a2a* mutant with a 15 and 11 bp deletion (net -26 bp) in exon 4 that removes the splice acceptor site and part of the exon (S8D Fig); we named this allele *c1001*. Incrossing *slc3a2a*^*c1001/*+^ fish produces larvae with the dark yolk phenotype (Fig 6D). The frequency of dark yolk in *c1001* was slightly sub-mendelian (17 ± 9%) suggesting the *c1001* allele may be more mild and not fully penetrant. Importantly, the *c1001* allele fails to complement *zion*, as outcrossing *slc3a2*^*c1001/*+^ to *zion*^+/-^ also produces dark yolk larvae in the predicted mendelian ratio (23 ± 5%) (Fig 6E). The same result was observed for two additional CRISPR alleles of *slc3a2a* (*c1040*, *c1041*) (S8E and S8F Fig).

The C > T mutation in *zion* mutants was confirmed by Sanger Sequencing of genomic DNA (S8G Fig) and a genotyping protocol was developed. *slc3a2a^zion^* larvae had significantly fewer B-lps after phenotype onset (4 dpf) relative to *slc3a2a*^+/*zion*^ and *slc3a2a*^+/+^ siblings (Fig 6F). The human ortholog of *slc3a2a*, SLC3A2, heterodimerizes with SLC7 family members to form amino acid exchangers [39]. To further evaluate the potential role for SLC3A2 in B-lp metabolism we evaluated SLC3A2 and SLC7 genes for polymorphisms associated with abnormal lipid metabolism parameters using the HUGE score calculator [40]. While SLC3A2 itself is not associated with dyslipidemic phenotypes, several of its binding partners (SLC7A2, SLC7A6, SLC7A10, and SLC7A13) are associated with altered plasma lipids in humans (Fig 6G). Taken together, these data illustrate the power and efficiency of the WheresWalker pipeline for mutation mapping.

## Discussion

Here we introduce WheresWalker, a mutation mapping protocol based on bulk segregant analysis and demonstrate its ability to identify multiple genetic variants, including 4 novel mutations responsible for dark yolk phenotypes in zebrafish. The WheresWalker software 1) calculates a SNP index based on decreased mutant heterozygosity to identify genomic regions linked to a mutant phenotype, 2) filters variants to generate a list of the most likely candidates, and 3) automatically identifies background polymorphisms that can be used for recombinant mapping to refine the computationally defined interval and narrow the list of candidate genes. In zebrafish, candidate genes can be rapidly tested using high-efficiency CRISPR/Cas9 reverse genetics. This hybrid model leverages the best of both traditional and contemporary approaches to enable rapid mutation mapping on the order of weeks, as opposed to years. We rigorously test the mapping-by-sequencing component of WheresWalker by identifying 3 loci from a recent mutagenesis screen in zebrafish, and show that WheresWalker can also be applied to map mutations in maize, and likely many other species. Consistent with modeling of sequencing coverage in Arabidopsis with the SHOREmap tool [18], we find the number of candidate mutations is reduced with increased coverage (Table 1). Based on our data (Table 1, Fig 3H), we recommend sequencing at ~30X coverage to identify a high-quality interval with the fewest SNP candidates.

In the zebrafish datasets, we observed substantial variability in the size of the mapped interval. We identified intervals as small as ~10 Mb and as large as ~ 55 Mb. For the *mttp*^*arches*^ mutant, we showed that using multiple parental pairs to generate genomic DNA for sequencing results in narrower intervals (Table 1, Fig 4E). For each of the *mttp*^*arches*^, *apobb.1*^*olympic*^, and *mia2*^*teton*^ mutants, F2 incrosses led to the smallest interval. The *apobb.1*^*olympic*^ interval was the largest zebrafish interval observed (>35 Mb), which may be due to the proximity of the locus to the centromere. Using F3, F4, etc. generations would allow for more recombination, and therefore narrower intervals, though we did not directly test this hypothesis. However, consistent with this hypothesis, we identified larger intervals when using parents outcrossed to WIK, which are “F1” in terms of recombination. Using WIK-outcrossed parents increased the SNP index around the causative locus, as did increasing the number of individuals represented in each bulk (Table 1, Fig 4A–4C and 4E). Factors that increase the genetic diversity (clutches represented, outcross status) and chances for recombination (generation, number of animals) should be considered when designing a WheresWalker mapping experiment.

We designed WheresWalker to be a more robust approach for identifying mutations in regions of low sequencing coverage, such as the near the centromere and in non-coding regions where sequence tends to be more repetitive. Instead of using allelic frequency, which is sensitive to sequencing coverage, our mapping algorithm considers the relative number of non-homozygous alleles in a genomic region, making it less sensitive to coverage variation. While we attempted to directly compare WheresWalker to previously published allelic frequency mapping tools, we were unable to find a functional tool that accepted the sequencing inputs available for our mutants. This underscores the need for an easy to deploy, open source positional cloning tool.

As we demonstrate with the *vns* maize mutant, the WheresWalker approach is better at identifying the causative chromosome than assessing the density of homozygous variants (S4 Fig). Mapping homozygous SNPs that are specific to the mutant background by using a sibling sequencing set to eliminate background homozygosity is a similar approach that was also tested. SNPs that are homozygous in the mutant sample but not the siblings are distributed across the genome but are concentrated on the causative chromosome. Homozygous density plots are similar to the WheresWalker SNPindex output and complement the WheresWalker analysis. However, because segregating homozygous SNPs are rare (40–2000 points/genome in our datasets), this approach may be particularly sensitive to factors that decrease the number of detectable homozygous points which would include sequencing coverage, phenotype missorting and background similarity to the selected genome assembly.

With any mapping-by-sequencing approach, larger intervals are expected to be selected near centromeres due to lower rates of recombination. These larger intervals can be refined using recombinant mapping markers outputted by WheresWalker, where flanking markers (in regions of higher recombination) can be used to point toward the genomic region containing the gene of interest. When known, local recombination rates can be used to predict the causative gene location most accurately. To our knowledge, WheresWalker is the first mapping tool which automates indel marker identification for recombinant mapping.

Using WheresWalker, we map a novel dark yolk mutant, *zion*, to a nonsense mutation in *slc3a2a* and show that this mutation leads to reduced levels of B-lp suggesting a defect in B-lp biogenesis. To our knowledge, there is currently no published literature linking *slc3a2a* directly to B-lp metabolism. SLC3A2 itself is not associated with human dyslipidemia, but several SLC7 dimer partners are, suggesting the link between *slc3a2a* function and B-lp metabolism may be specific to a subset of transported substrates (e.g., amino acids), tissue expression patterns, and/or subcellular localization. Amino acid availability may play a direct role in B-lp synthesis by providing substrate for the synthesis of ApoB (a 4563 aa protein in humans and a 3730 aa protein in zebrafish). Many amino acids (Ala, Arg, Asn, Gln, His, Leu, Met, Ser, Thr, and Val) activate mTORC1 activity [41]. mTORC1 activity is associated with increased secretion of B-lps [42,43] and has been linked to metabolic disease states [44,45]. Specifically, the SLC3A2/SLC7A5 leucine transporter has been shown to be important for mTORC1 activation [46]. Further characterization will be required to better understand the mechanistic role of SLC3A2 in B-lp metabolism. These data demonstrate the power of unbiased forward genetic approaches to assign new functions to genes.

CRISPR has emerged as a powerful tool for reverse genetics, but has increasingly been used for genome-wide screening to generate null alleles (CRISPRko) [19,47–49]. However, forward genetic ENU/EMS mutagenesis screens remain relevant for their propensity to generate null as well as gain-of-function and partial-function alleles, a functionality that is not yet available for high-throughput CRISPR-based technology. Moreover, chemical mutagenesis can be deployed in organisms for which CRISPR or other mutagenesis strategies have not been developed. Historically, the major drawback of chemical mutagenesis approaches has been the years required to identify the gene/causative mutation(s) underlying exciting new phenotypes. Many of the existing mapping-by-sequencing tools are deprecated and require bioinformatics expertise to fix and use them. Beyond this, existing tools do not have an integrated indel mapping component. WheresWalker solves this problem. It is an efficient, easy-to-use, and freely available mapping and positional cloning pipeline that requires virtually no bioinformatics experience. Because WheresWalker utilizes background polymorphisms for interval picking and fine mapping, it is best deployed in organisms bred in polygenic backgrounds. In the current wave of emerging model organisms, we anticipate WheresWalker will be a critical tool for foundational mutagenesis screens in novel model species and can also be applied to yet unmapped mutants and modifiers from historical and contemporary screens in traditional genetic models.

### Limitations of WheresWalker

WheresWalker is unlikely to be as effective in organisms with low polymorphism or without a divergent strain for outcrossing. Low heterogeneity blunts the SNPindex and limits the number of markers available for mapping, making it difficult to identify causative mutations in highly homozygous regions. As we have shown, zebrafish have sufficient polymorphism in the AB strain to support mapping. But when this is not the case, polymorphism can also be introduced by outcrossing to an alternative background. Polymorphism helps to define the interval, but also provides markers for recombinant mapping. Markers are sometimes located in regions of low sequence complexity making it difficult to design unique primers. For this reason, some indels can not be assessed. In our datasets, we observe hundreds of indels, so this is unlikely to be prohibitive in the zebrafish system. WheresWalker is currently built to identify candidates by filtering for single base pair mutations, but could be adapted to identify different types of mutations. Finally, non-coding regions remain poorly annotated and understood. While WheresWalker is able to consider these mutations using WGS, prioritization of functional mutations remains a challenge to the field.

## Supporting information

S1 Fig Phenotype frequency and complementation for additional novel DY alleles. A) Phenotype frequency for all mutants identified. Clutches/fish for each mutant: c897:11/634, c898: 8/456, c899: 6/405, c900: NA, c901: 9/701, c902: 5/161, c903: 9/627, c904: 5/398, c905: 6/529, c907: 8/589, c908: 4/399, c909: 7/566, c910: 7/541, c911: 5/534, c912: 7/534, c913: 4/228, c914: 4/218, c917: 5/389, c918: 17/1495, c919: 1/47, c920: 2/199, c923: 7/417, c924: 3/151, c952: 6/304, c926: 2/194, c929: 4/364, c930: 4/400, c931: 7/650. Bars represent mean ± standard deviation. Dashed line indicates the expected frequency of 0.25. B-E) Representative images for 7 additional mutants that fail to complement known dark yolk loci, including 3 *mttp* (B), 2 *dgat2* (C), 1 *mia2* (D), and 1 *apobb.1* (E) alleles. Representative wild-type (wt) and mutant (mut) yolk phenotypes are shown. Animal age is noted. Phenotype frequency is reported as mean ± standard deviation. *bigbend*: N = 5 clutches, n = 422 animals; *guadalupe*: N = 6 clutches, n = 598 animals; *carlsbad*: N = 8 clutches, n = 784 animals; *rainier*: N = 3 clutches, n = 210 animals; *cascade*: N = 4 clutches, n = 402 animals; *mammoth*: N = 3 clutches, n = 282 animals; *dune*: N = 4 clutches, n = 180 animals;. Scale bar represents 1 mm.
(TIFF)

S2 Fig SNP Index profile for all chromosomes corresponding to regional plots in Figs 3 and 4. Solid lines indicate left and right bounds of the interval selected by WheresWalker, dashed lines indicate the position of the causative locus.
(TIFF)

S3 Fig Het^wt^ distribution for *arches* WIK 1x28, 3x10, 4x5, and n = 50 datasets. Binwidth is 1. Dashed lines mark the median (1x28: 12.80365, 3x10: 13.5894, 4x5: 13.81362, n = 50: 28.94921).
(TIFF)

S4 Fig WheresWalker outperforms homozygosity density mapping for Maize mutant. VCF files were generated using POLCA for mutant and sibling datasets for the *vns* maize mutants. A) points of homozygosity that differ from the reference genome at a frequency >0.9 in the mutant dataset were identified and the density of these points across each chromosome was plotted. Both NC_050096.1 and NC_050104.1 have regions with a high density of homozygous SNPs. B) mutant and sibling VCF files were submitted to WheresWalker. The SNP index across all chromosomes is plotted. WheresWalker identifies a single interval containing the causative gene, *dek1* (LOC542509). C) Het^wt^ distribution. Binwidth is 1. Dashed line indicates the mean Het^wt^ (4.642325).
(TIFF)

S5 Fig Bulk Segregant Analysis (BSA) homozygosity mapping mirrors WheresWalker output. A) Distribution of BSA filtered homozygous points across the genome for *mttp*^*arches*^, *apobb.1*^*olympic*^, and *mia2*^*teton*^ mutants in the AB background. B-D) Density plots of BSA filtered homozygous points (top) and WheresWalker output (bottom) for the causative chromosome for *mttp*^*arches*^ (B, chr01), *apobb.1*^*olympic*^ (C, chr20), and *mia2*^*teton*^ (D, chr17).
(TIFF)

S6 Fig Mapping and confirmation of the *arches* locus. A) genotyping gels for markers A_a_ and B_a_ for *arches* mutants 9–35. PCR product sizes for wild-type and mutant (highlighted) products are as indicated. B) Sanger Sequencing of normal (NY) and dark yolk (DY) animals from an *mttp*^*arches/*+^ in-cross have the expected genotypes at the mutant position: wild-type (wt) - C, heterozygous (het) - C/A, mutant (mut) - A.
(TIFF)

S7 Fig Mapping of the *zion* locus. A) recombination gel for *zion* parents (M: male, F: Female) and selected *zion* mutant progeny at markers A_z_-E_z_. Genotype score is as indicated above each lane. PCR product sizes for wild-type and mutant (highlighted) products are shown. B) Marker genotype score for all *zion* mutant progeny. Green shading indicates mutant genotype, gray shading indicates heterozygous genotype, white background indicates genotype could not be determined. Outlined boxes with “?” indicate the genotype could not be determined by gel, but could be inferred based on the genotype of the surrounding markers for that animal. C) Summary of genotype at each marker with calculations for recombination frequency (Rf) and estimated distance to mutation calculated with and without inferred genotypes.
(TIFF)

S8 Fig Confirmation of the *zion* locus. A) Schematic of the *slc3a2a* and *slc3a2b* genes. The location of the C > T base pair change in *slc3a2a* in the *zion* mutants is shown in purple. Red carats indicate locations targeted by CRISPR guides. B-C) PCR amplification around CRISPR guide sites in *slc3a2a* (B) and *slc3a2b* (C) injected animals. An uninjected animal (UI) was genotyped for comparison. “g#” indicates the guide site that is amplified for the respective gene, the expected size for each product is indicated on the right of each gel. For *slc3a2a*, editing is observed in many animals at g1 and g2/3; for *slc3a2b*, editing is observed in many animals at g4. D) *c1001* (-15 and -11 bp), *c1040* (-9 and -3 bp), and *c1041* (-11 + 5 bp) *slc3a2a* alleles exhibit insertions/deletions in exon 4 and the exon 4 splice site as determined by Sanger Sequencing; exon 4 is highlighted in green. E) In-crossing *slc3a2a*^*c1040/*+^ (N = 2, n = 558) or *slc3a2a*^*c1041/*+^ (N = 2, n = 387) CRISPR mutants produces offspring with dark yolk. F) Outcrossing *zion*^+/-^ to *slc3a2a*^*c1040/*+^ (N = 3, n = 299) or *slc3a2a*^*c1041/*+^ (N = 3, n = 219) produces offspring with dark yolk. For E-F, animals are 5 dpf, dark yolk frequency is reported as mean ± standard deviation, scale bar represents 1 mm. G) Sanger Sequencing of normal (NY) and dark yolk (DY) animals from a *zion*^+/-^ in-cross have the expected genotype at the mutant position: wild-type (wt) - C, heterozygous (het) - C/T, mutant (mut) - T.
(TIFF)

S1 File Supplementary methods. (DOCX)

S2 File Oligo sequences. (XLSX)

S3 File SRA accession numbers for BioProject PRJNA1187516. (XLSX)

S4 File Mapping indices: WheresWalker SNP index, Het^wt^, and homozygosity values for zebrafish and maize datasets. (XLSX)

S5 File Candidate lists generated by WheresWalker for each mutant. (XLSX)

S6 File – Indel markers generated by WheresWalker for each mutant. (XLSX)

S7 File Phenotype frequencies for each mutant and for complementation testing. (XLSX)

S8 File Raw data values for ApoBb.1-nanoluciferase luminescence measurements. (XLSX)

## References

[1] Rocha JJ, Jayaram SA, Stevens TJ, Muschalik N, Shah RD, Emran S, et al. Functional unknomics: systematic screening of conserved genes of unknown function. PLoS Biol. 2023;21(8):e3002222. doi: 10.1371/journal.pbio.3002222 37552676 PMC10409296

[2] Zhou Y, Zon LI. The Zon laboratory guide to positional cloning in zebrafish. In: Detrich HW, Westerfield M, Zon LI, editors. Methods in cell biology. Academic Press; 2011. p. 287–309. 10.1016/B978-0-12-374814-0.00016-1 21924169

[3] Hill JT, Demarest BL, Bisgrove BW, Gorsi B, Su YC, Yost HJ. MMAPPR: mutation mapping analysis pipeline for pooled RNA-seq. Genome Res. 2013;23:687–97. 23299975 10.1101/gr.146936.112 PMC3613585

[4] Gray RS, Gonzalez R, Ackerman SD, Minowa R, Griest JF, Bayrak MN. Postembryonic screen for mutations affecting spine development in zebrafish. Dev Biol. 2021;471:18–33. 33290818 10.1016/j.ydbio.2020.11.009 PMC10785604

[5] Schneeberger K, Ossowski S, Lanz C, Juul T, Petersen AH, Nielsen KL, et al. SHOREmap: simultaneous mapping and mutation identification by deep sequencing. Nat Methods. 2009;6(8):550–1. doi: 10.1038/nmeth0809-550 19644454

[6] Obholzer N, Swinburne IA, Schwab E, Nechiporuk AV, Nicolson T, Megason SG. Rapid positional cloning of zebrafish mutations by linkage and homozygosity mapping using whole-genome sequencing. Development. 2012;139(22):4280–90. doi: 10.1242/dev.083931 23052906 PMC3478692

[7] Lup SD, Wilson-Sánchez D, Andreu-Sánchez S, Micol JL. Easymap: a user-friendly software package for rapid mapping-by-sequencing of point mutations and large insertions. Front Plant Sci. 2021;12:655286. 34040621 10.3389/fpls.2021.655286 PMC8143052

[8] Abe A, Kosugi S, Yoshida K, Natsume S, Takagi H, Kanzaki H, et al. Genome sequencing reveals agronomically important loci in rice using MutMap. Nat Biotechnol. 2012;30(2):174–8. doi: 10.1038/nbt.2095 22267009

[9] Minevich G, Park DS, Blankenberg D, Poole RJ, Hobert O. CloudMap: a cloud-based pipeline for analysis of mutant genome sequences. Genetics. 2012;192(4):1249–69. doi: 10.1534/genetics.112.144204 23051646 PMC3512137

[10] Wachsman G, Modliszewski JL, Valdes M, Benfey PN. A simple pipeline for mapping point mutations. Plant Physiol. 2017;174:1307–13. 28546435 10.1104/pp.17.00415 PMC5490893

[11] Leshchiner I, Alexa K, Kelsey P, Adzhubei I, Austin-Tse CA, Cooney JD, et al. Mutation mapping and identification by whole-genome sequencing. Genome Res. 2012;22(8):1541–8. doi: 10.1101/gr.135541.111 22555591 PMC3409267

[12] Bowen ME, Henke K, Siegfried KR, Warman ML, Harris MP. Efficient mapping and cloning of mutations in zebrafish by low-coverage whole-genome sequencing. Genetics. 2012;190(3):1017–24. doi: 10.1534/genetics.111.136069 22174069 PMC3296239

[13] Henke K, Bowen ME, Harris MP. Perspectives for identification of mutations in the zebrafish: making use of next-generation sequencing technologies for forward genetic approaches. Methods. 2013;62(3):185–96. doi: 10.1016/j.ymeth.2013.05.015 23748111

[14] Wolman MA, Jain RA, Marsden KC, Bell H, Skinner J, Hayer KE, et al. A genome-wide screen identifies PAPP-AA-mediated IGFR signaling as a novel regulator of habituation learning. Neuron. 2015;85(6):1200–11. doi: 10.1016/j.neuron.2015.02.025 25754827 PMC4368495

[15] Lup SD, Navarro-Quiles C, Micol JL. Versatile mapping-by-sequencing with Easymap v.2. Front Plant Sci. 2023;14:1042913. doi: 10.3389/fpls.2023.1042913 36778692 PMC9909543

[16] Sun H, Schneeberger K. SHOREmap v3.0: fast and accurate identification of causal mutations from forward genetic screens. Methods Mol Biol. 2015;1284:381–95. 25757783 10.1007/978-1-4939-2444-8_19

[17] Sugihara Y, Young L, Yaegashi H, Natsume S, Shea DJ, Takagi H, et al. High-performance pipeline for MutMap and QTL-seq. PeerJ. 2022;10:e13170. doi: 10.7717/peerj.13170 35321412 PMC8935991

[18] James GV, Patel V, Nordström KJV, Klasen JR, Salomé PA, Weigel D, et al. User guide for mapping-by-sequencing in Arabidopsis. Genome Biol. 2013;14(6):R61. doi: 10.1186/gb-2013-14-6-r61 23773572 PMC3706810

[19] Wu RS, Lam II, Clay H, Duong DN, Deo RC, Coughlin SR. A rapid method for directed gene knockout for screening in G0 zebrafish. Dev Cell. 2018;46(1):112-125.e4. doi: 10.1016/j.devcel.2018.06.003 29974860

[20] Guryev V, Koudijs MJ, Berezikov E, Johnson SL, Plasterk RHA, van Eeden FJM. Genetic variation in the zebrafish. Genome Res. 2006;16:491–7. 16533913 10.1101/gr.4791006 PMC1457036

[21] Zimin AV, Salzberg SL. The genome polishing tool POLCA makes fast and accurate corrections in genome assemblies. PLoS Comput Biol. 2020;16(6):e1007981. doi: 10.1371/journal.pcbi.1007981 32589667 PMC7347232

[22] Wang K, Li M, Hakonarson H. ANNOVAR: functional annotation of genetic variants from high-throughput sequencing data. Nucleic Acids Res. 2010;38(16):e164. doi: 10.1093/nar/gkq603 20601685 PMC2938201

[23] Ng PC, Henikoff S. SIFT: predicting amino acid changes that affect protein function. Nucleic Acids Res. 2003;31(13):3812–4. doi: 10.1093/nar/gkg509 12824425 PMC168916

[24] Shimoda N, Knapik EW, Ziniti J, Sim C, Yamada E, Kaplan S, et al. Zebrafish genetic map with 2000 microsatellite markers. Genomics. 1999;58(3):219–32. doi: 10.1006/geno.1999.5824 10373319

[25] Chew NWS, Ng CH, Tan DJH, Kong G, Lin C, Chin YH, et al. The global burden of metabolic disease: data from 2000 to 2019. Cell Metab. 2023;35(3):414-428.e3. doi: 10.1016/j.cmet.2023.02.003 36889281

[26] Santos AJM, Nogueira C, Ortega-Bellido M, Malhotra V. TANGO1 and Mia2/cTAGE5 (TALI) cooperate to export bulky pre-chylomicrons/VLDLs from the endoplasmic reticulum. J Cell Biol. 2016;213(3):343–54. doi: 10.1083/jcb.201603072 27138255 PMC4862334

[27] Templehof H, Moshe N, Avraham-Davidi I, Yaniv K. Zebrafish mutants provide insights into Apolipoprotein B functions during embryonic development and pathological conditions. JCI Insight. 2021;6(13):e130399. doi: 10.1172/jci.insight.130399 34236046 PMC8410079

[28] Wilson MH, Rajan S, Danoff A, White RJ, Hensley MR, Quinlivan VH. A point mutation decouples the lipid transfer activities of microsomal triglyceride transfer protein. PLoS Genet. 2020;16:e1008941. 10.1371/journal.pgen.1008941 PMC7444587 32760060

[29] Clark EM, Link BA. Complementary and divergent functions of zebrafish Tango1 and Ctage5 in tissue development and homeostasis. Mol Biol Cell. 2021;32(5):391–401. doi: 10.1091/mbc.E20-11-0745 33439675 PMC8098853

[30] Mullins MC, Hammerschmidt M, Haffter P, Nüsslein-Volhard C. Large-scale mutagenesis in the zebrafish: in search of genes controlling development in a vertebrate. Curr Biol. 1994;4(3):189–202. doi: 10.1016/s0960-9822(00)00048-8 7922324

[31] Thierer JH, Ekker SC, Farber SA. The LipoGlo reporter system for sensitive and specific monitoring of atherogenic lipoproteins. Nat Commun. 2019;10(1):3426. doi: 10.1038/s41467-019-11259-w 31366908 PMC6668417

[32] Wilson MH, Hensley MR, Shen M-C, Lu H-Y, Quinlivan VH, Busch-Nentwich EM, et al. Zebrafish are resilient to the loss of major diacylglycerol acyltransferase enzymes. J Biol Chem. 2024;300(12):107973. doi: 10.1016/j.jbc.2024.107973 39510175 PMC11663968

[33] Thierer JH, Foresti O, Yadav PK, Wilson MH, Moll TOC, Shen M-C, et al. Pla2g12b drives expansion of triglyceride-rich lipoproteins. Nat Commun. 2024;15(1):2095. doi: 10.1038/s41467-024-46102-4 38453914 PMC10920679

[34] Klein H, Xiao Y, Conklin PA, Govindarajulu R, Kelly JA, Scanlon MJ, et al. Bulked-segregant analysis coupled to whole genome sequencing (BSA-Seq) for rapid gene cloning in maize. G3 (Bethesda). 2018;8(11):3583–92. doi: 10.1534/g3.118.200499 30194092 PMC6222591

[35] Ricci B, Sharp D, O’Rourke E, Kienzle B, Blinderman L, Gordon D. A 30-amino acid truncation of the microsomal triglyceride transfer protein large subunit disrupts its interaction with protein disulfide-isomerase and causes abetalipoproteinemia *. J Biol Chem. 1995;270:14281–5. 7782284 10.1074/jbc.270.24.14281

[36] Otis JP, Zeituni EM, Thierer JH, Anderson JL, Brown AC, Boehm ED, et al. Zebrafish as a model for apolipoprotein biology: comprehensive expression analysis and a role for ApoA-IV in regulating food intake. Dis Model Mech. 2015;8(3):295–309. doi: 10.1242/dmm.018754 25633982 PMC4348566

[37] Miyares RL, de Rezende VB, Farber SA. Zebrafish yolk lipid processing: a tractable tool for the study of vertebrate lipid transport and metabolism. Dis Model Mech. 2014;7(7):915–27. doi: 10.1242/dmm.015800 24812437 PMC4073280

[38] Schlegel A, Stainier DYR. Microsomal triglyceride transfer protein is required for yolk lipid utilization and absorption of dietary lipids in zebrafish larvae. Biochemistry. 2006;45(51):15179–87. doi: 10.1021/bi0619268 17176039

[39] Fairweather SJ, Shah N, Brӧer S. Heteromeric solute carriers: function, structure, pathology and pharmacology. In: Atassi MZ, editor. Protein reviews: volume 21. Cham: Springer International Publishing; 2021. p. 13–127. 10.1007/5584_2020_584 33052588

[40] Dornbos P, Singh P, Jang D-K, Mahajan A, Biddinger SB, Rotter JI, et al. Evaluating human genetic support for hypothesized metabolic disease genes. Cell Metab. 2022;34(5):661–6. doi: 10.1016/j.cmet.2022.03.011 35421386 PMC9166611

[41] Meng D, Yang Q, Wang H, Melick CH, Navlani R, Frank AR, et al. Glutamine and asparagine activate mTORC1 independently of Rag GTPases. J Biol Chem. 2020;295(10):2890–9. doi: 10.1074/jbc.AC119.011578 32019866 PMC7062167

[42] Roberts JL, He B, Erickson A, Moreau R. Improvement of mTORC1-driven overproduction of apoB-containing triacylglyceride-rich lipoproteins by short-chain fatty acids, 4-phenylbutyric acid and (R)-α-lipoic acid, in human hepatocellular carcinoma cells. Biochim Biophys Acta. 2016;1861(3):166–76. doi: 10.1016/j.bbalip.2015.12.001 26680362

[43] Quinn WJ 3rd, Wan M, Shewale SV, Gelfer R, Rader DJ, Birnbaum MJ, et al. mTORC1 stimulates phosphatidylcholine synthesis to promote triglyceride secretion. J Clin Invest. 2017;127(11):4207–15. doi: 10.1172/JCI96036 29035283 PMC5663357

[44] Saxton RA, Sabatini DM. mTOR signaling in growth, metabolism, and disease. Cell. 2017;168(6):960–76. doi: 10.1016/j.cell.2017.02.004 28283069 PMC5394987

[45] Khamzina L, Veilleux A, Bergeron S, Marette A. Increased activation of the mammalian target of rapamycin pathway in liver and skeletal muscle of obese rats: possible involvement in obesity-linked insulin resistance. Endocrinology. 2005;146(3):1473–81. doi: 10.1210/en.2004-0921 15604215

[46] Nicklin P, Bergman P, Zhang B, Triantafellow E, Wang H, Nyfeler B, et al. Bidirectional transport of amino acids regulates mTOR and autophagy. Cell. 2009;136(3):521–34. doi: 10.1016/j.cell.2008.11.044 19203585 PMC3733119

[47] Bhattacharya D, Marfo CA, Li D, Lane M, Khokha MK. CRISPR/Cas9: an inexpensive, efficient loss of function tool to screen human disease genes in Xenopus. Dev Biol. 2015;408(2):196–204. doi: 10.1016/j.ydbio.2015.11.003 26546975 PMC4684459

[48] Bower OJ, McCarthy A, Lea RA, Alanis-Lobato G, Zohren J, Gerri C, et al. Generating CRISPR-Cas9-Mediated null mutations and screening targeting efficiency in human pluripotent stem cells. Curr Protoc. 2021;1(8):e232. doi: 10.1002/cpz1.232 34432381

[49] Bock C, Datlinger P, Chardon F, Coelho MA, Dong MB, Lawson KA, et al. High-content CRISPR screening. Nat Rev Methods Primers. 2022;2(1):9. doi: 10.1038/s43586-022-00098-7 37214176 PMC10200264

