# Supplementary figures and images for: Phenotype to genotype: A new and rapid approach using whole-genome sequencing
Source: PLoS Genet. 2025 Jul 14;21(7):e1011702. doi: 10.1371/journal.pgen.1011702 (PMC12273961; doi:10.1371/journal.pgen.1011702)

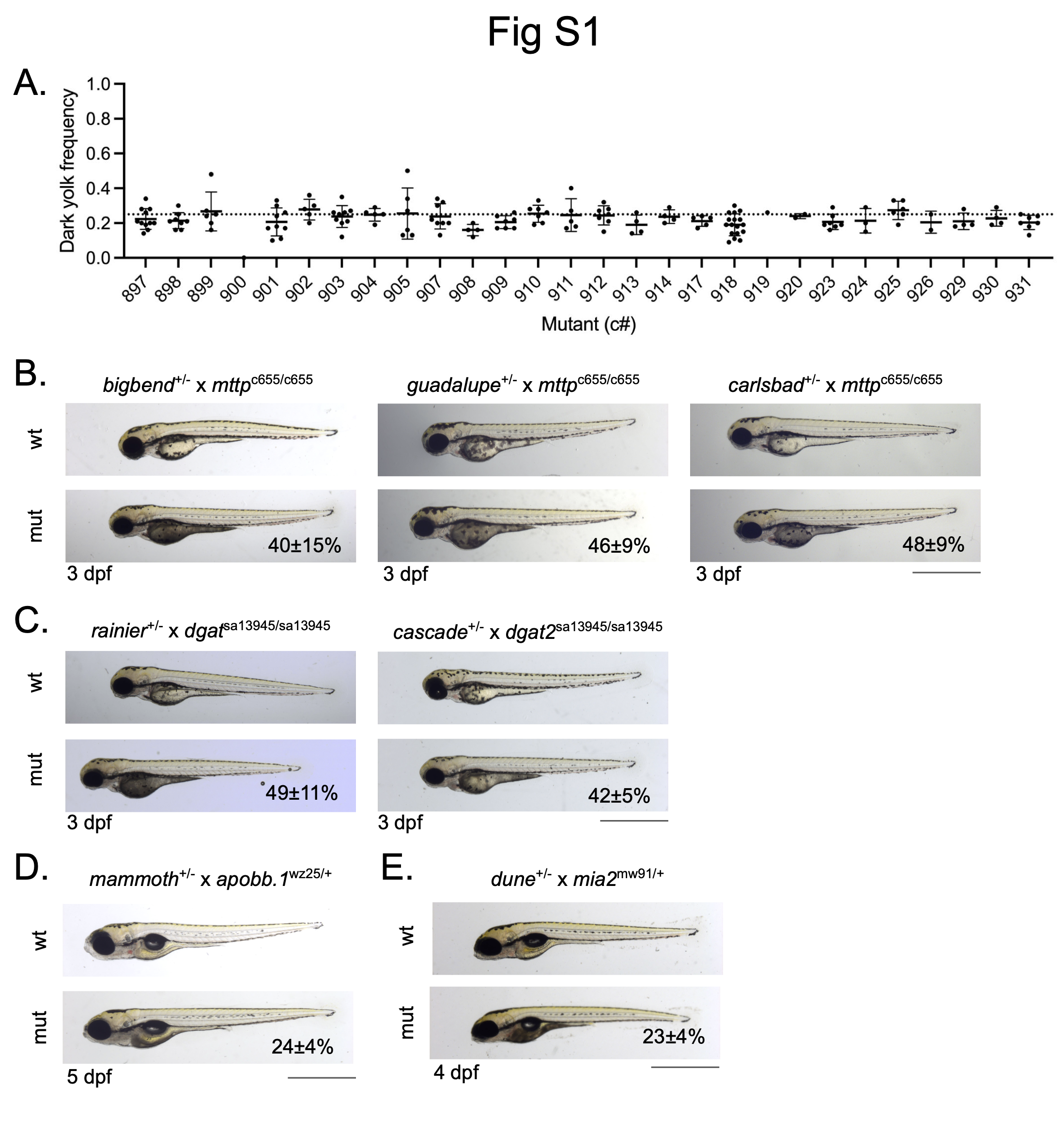

Supplement: S1 Fig — A) Phenotype frequency for all mutants identified. Clutches/fish for each mutant: c897:11/634, c898: 8/456, c899: 6/405, c900: NA, c901: 9/701, c902: 5/161, c903: 9/627, c904: 5/398, c905: 6/529, c907: 8/589, c908: 4/399, c909: 7/566, c910: 7/541, c911: 5/534, c912: 7/534, c913: 4/228, c914: 4/218, c917: 5/389, c918: 17/1495, c919: 1/47, c920: 2/199, c923: 7/417, c924: 3/151, c952: 6/304, c926: 2/194, c929: 4/364, c930: 4/400, c931: 7/650. Bars represent mean ± standard deviation. Dashed line indicates the expected frequency of 0.25. B-E) Representative images for 7 additional mutants that fail to complement known dark yolk loci, including 3 mttp (B), 2 dgat2 (C), 1 mia2 (D), and 1 apobb.1 (E) alleles. Representative wild-type (wt) and mutant (mut) yolk phenotypes are shown. Animal age is noted. Phenotype frequency is reported as mean ± standard deviation. bigbend: N = 5 clutches, n = 422 animals; guadalupe: N = 6 clutches, n = 598 animals; carlsbad: N = 8 clutches, n = 784 animals; rainier: N = 3 clutches, n = 210 animals; cascade: N = 4 clutches, n = 402 animals; mammoth: N = 3 clutches, n = 282 animals; dune: N = 4 clutches, n = 180 animals;. Scale bar represents 1 mm. (TIFF) [file pgen.1011702.s001.tiff]

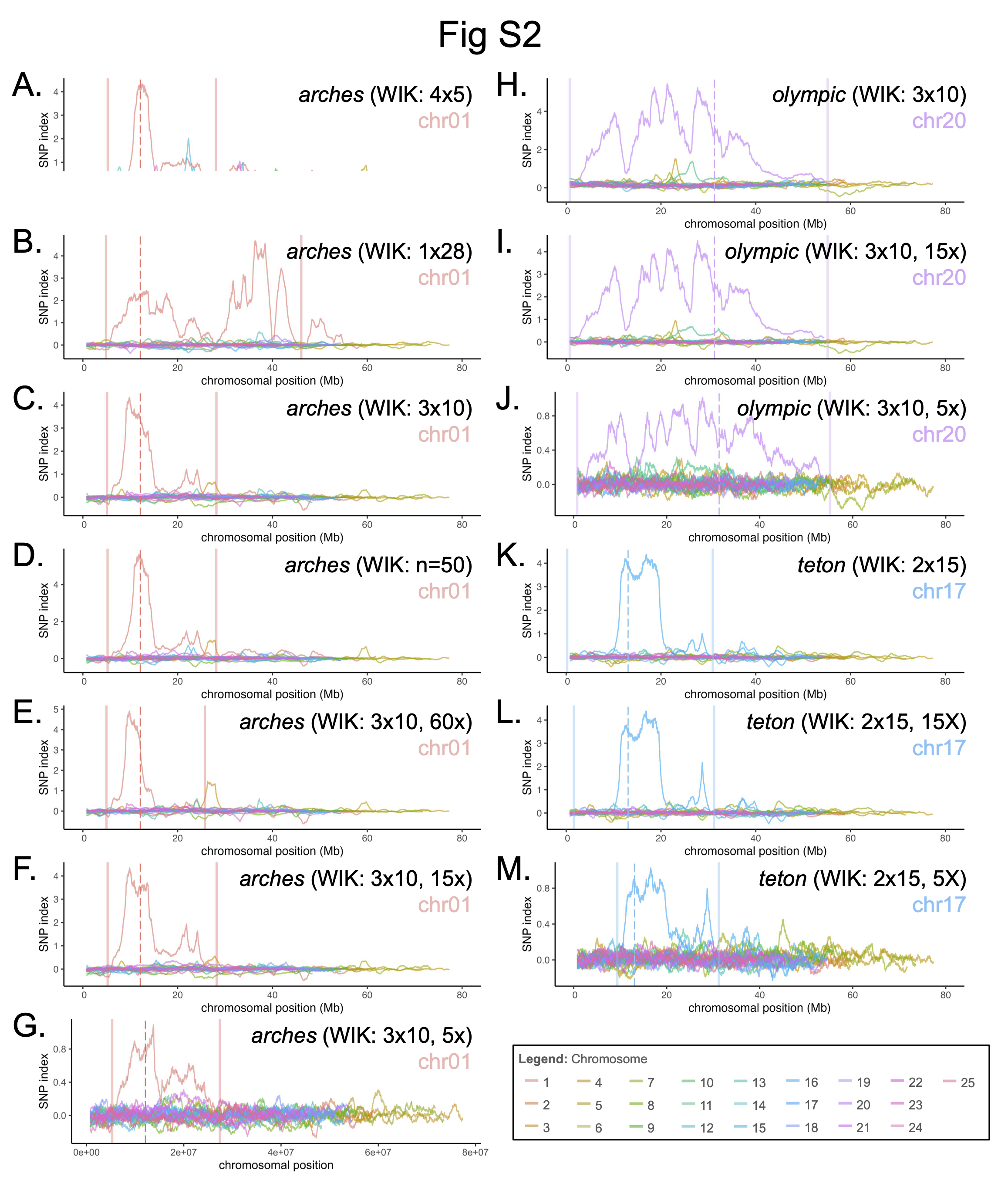

Supplement: S2 Fig — Solid lines indicate left and right bounds of the interval selected by WheresWalker, dashed lines indicate the position of the causative locus. (TIFF) [file pgen.1011702.s002.tiff]

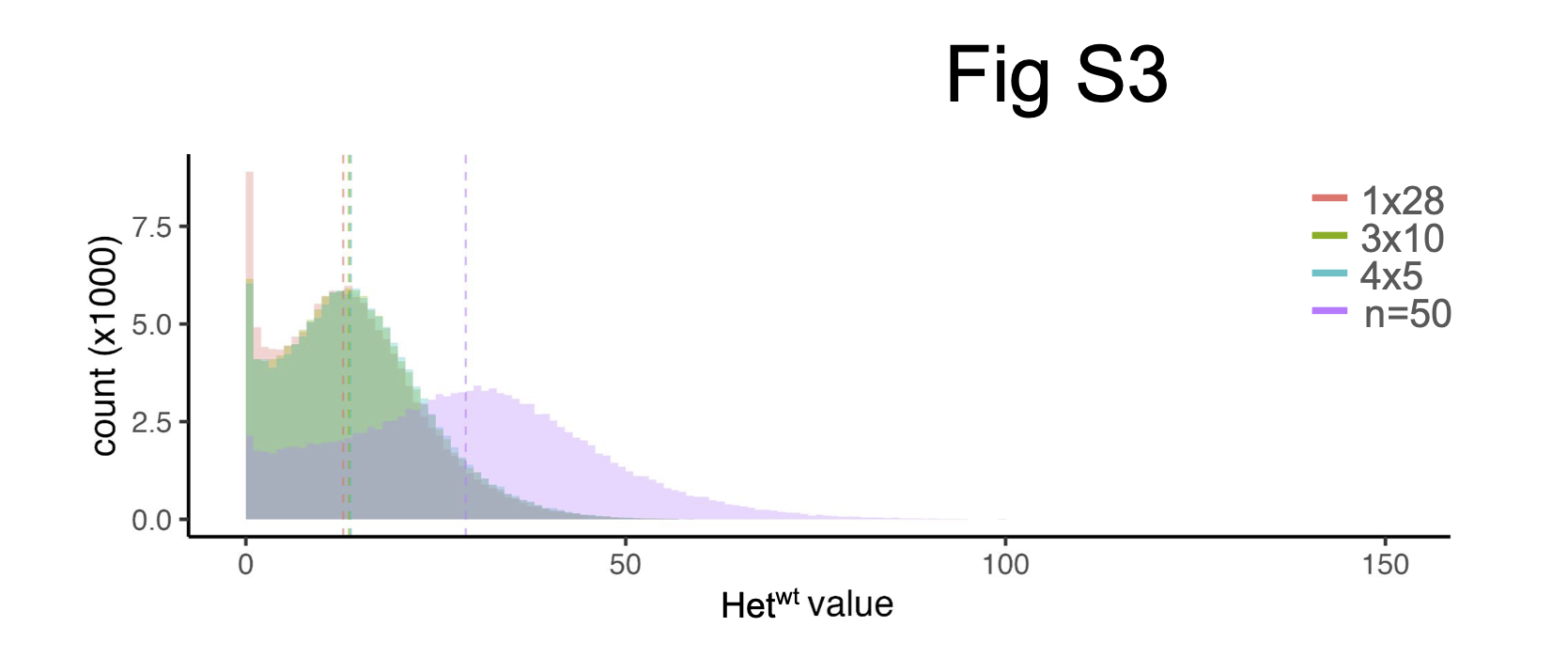

Supplement: S3 Fig — Binwidth is 1. Dashed lines mark the median (1x28: 12.80365, 3x10: 13.5894, 4x5: 13.81362, n = 50: 28.94921). (TIFF) [file pgen.1011702.s003.tiff]

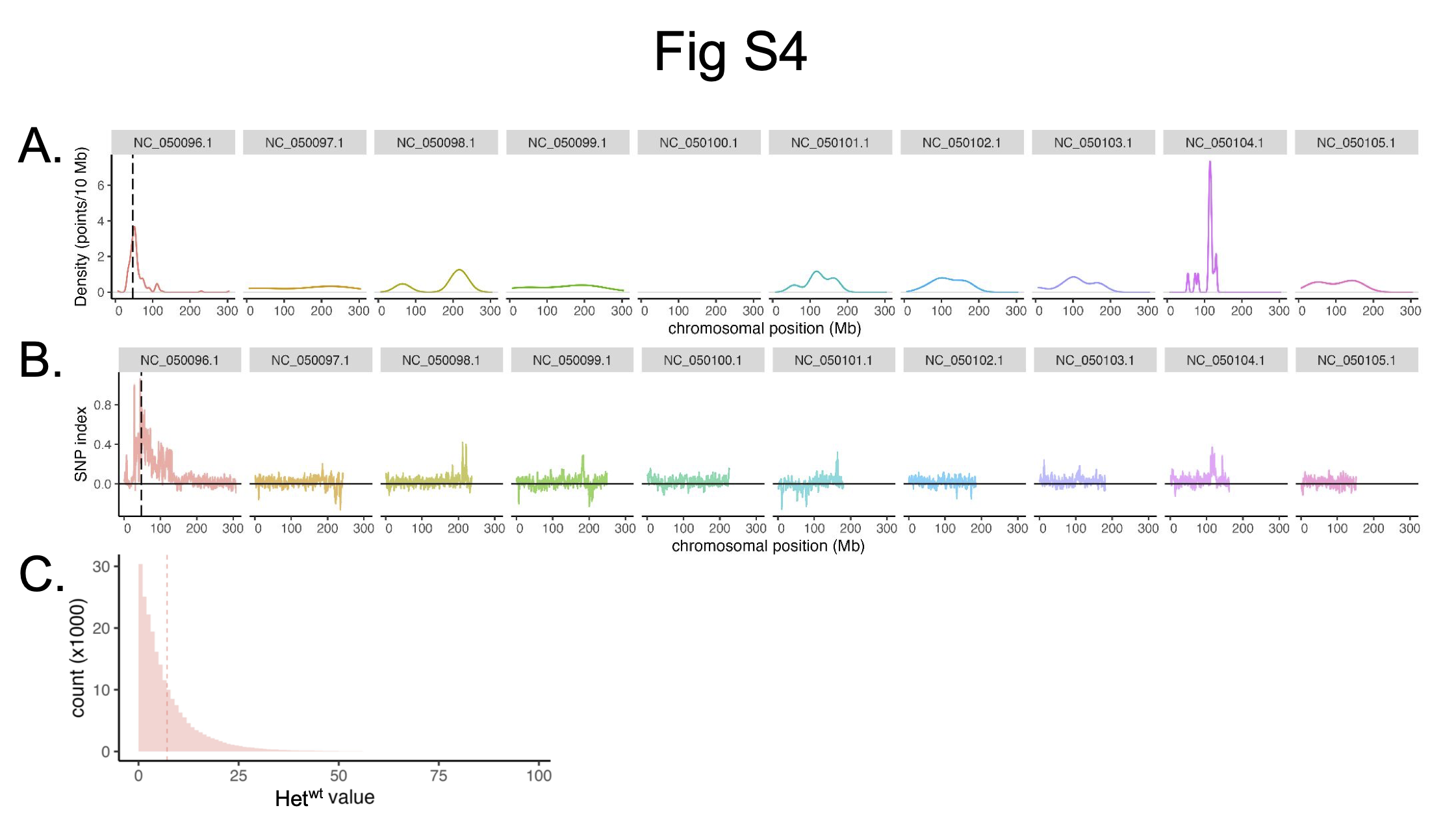

Supplement: S4 Fig — VCF files were generated using POLCA for mutant and sibling datasets for the vns maize mutants. A) points of homozygosity that differ from the reference genome at a frequency >0.9 in the mutant dataset were identified and the density of these points across each chromosome was plotted. Both NC_050096.1 and NC_050104.1 have regions with a high density of homozygous SNPs. B) mutant and sibling VCF files were submitted to WheresWalker. The SNP index across all chromosomes is plotted. WheresWalker identifies a single interval containing the causative gene, dek1 (LOC542509). C) Hetwt distribution. Binwidth is 1. Dashed line indicates the mean Hetwt (4.642325). (TIFF) [file pgen.1011702.s004.tiff]

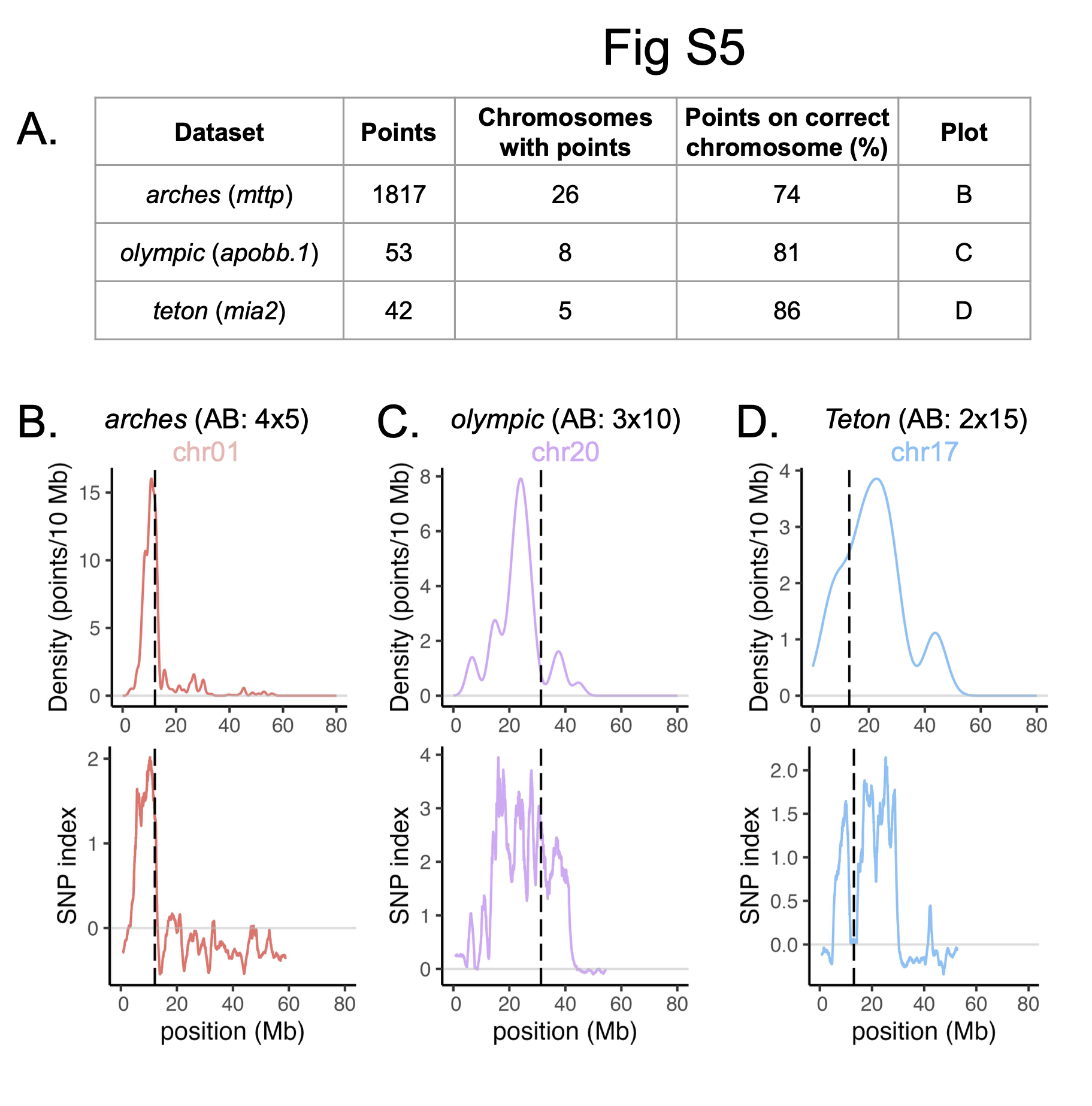

Supplement: S5 Fig — A) Distribution of BSA filtered homozygous points across the genome for mttparches, apobb.1olympic, and mia2teton mutants in the AB background. B-D) Density plots of BSA filtered homozygous points (top) and WheresWalker output (bottom) for the causative chromosome for mttparches (B, chr01), apobb.1olympic (C, chr20), and mia2teton (D, chr17). (TIFF) [file pgen.1011702.s005.tiff]

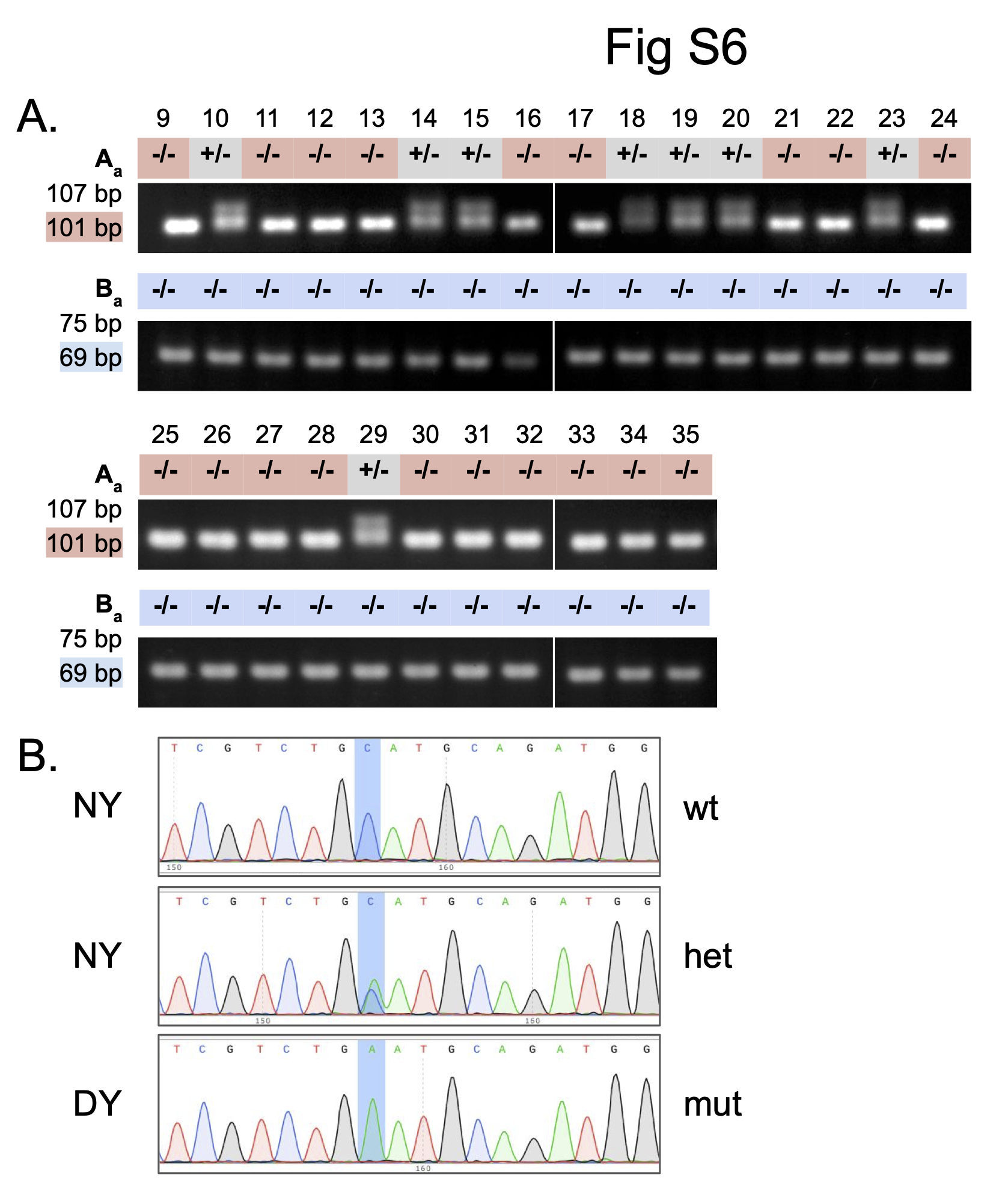

Supplement: S6 Fig — A) genotyping gels for markers Aa and Ba for arches mutants 9–35. PCR product sizes for wild-type and mutant (highlighted) products are as indicated. B) Sanger Sequencing of normal (NY) and dark yolk (DY) animals from an mttparches/+ in-cross have the expected genotypes at the mutant position: wild-type (wt) - C, heterozygous (het) - C/A, mutant (mut) - A. (TIFF) [file pgen.1011702.s006.tiff]

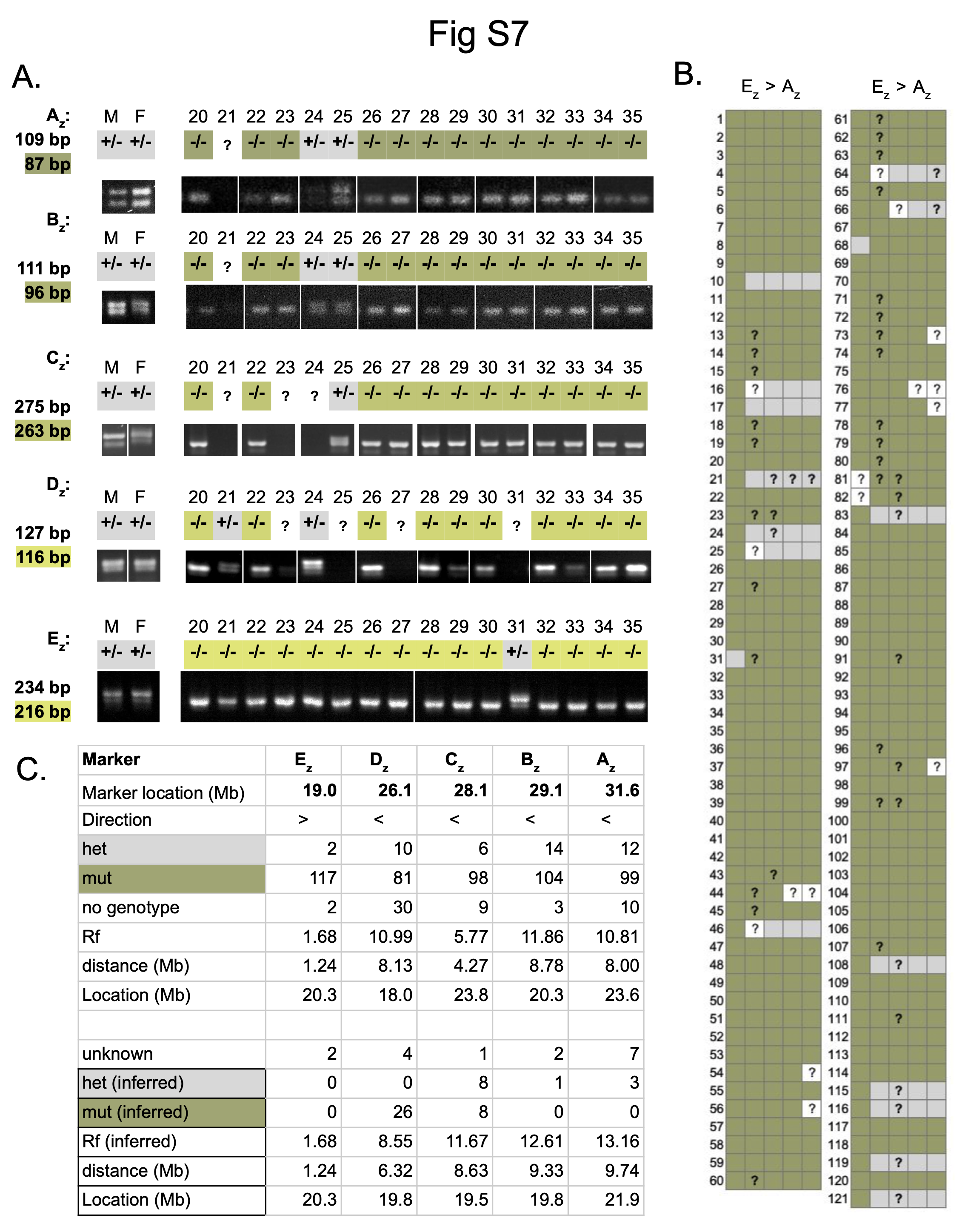

Supplement: S7 Fig — A) recombination gel for zion parents (M: male, F: Female) and selected zion mutant progeny at markers Az-Ez. Genotype score is as indicated above each lane. PCR product sizes for wild-type and mutant (highlighted) products are shown. B) Marker genotype score for all zion mutant progeny. Green shading indicates mutant genotype, gray shading indicates heterozygous genotype, white background indicates genotype could not be determined. Outlined boxes with “?” indicate the genotype could not be determined by gel, but could be inferred based on the genotype of the surrounding markers for that animal. C) Summary of genotype at each marker with calculations for recombination frequency (Rf) and estimated distance to mutation calculated with and without inferred genotypes. (TIFF) [file pgen.1011702.s007.tiff]

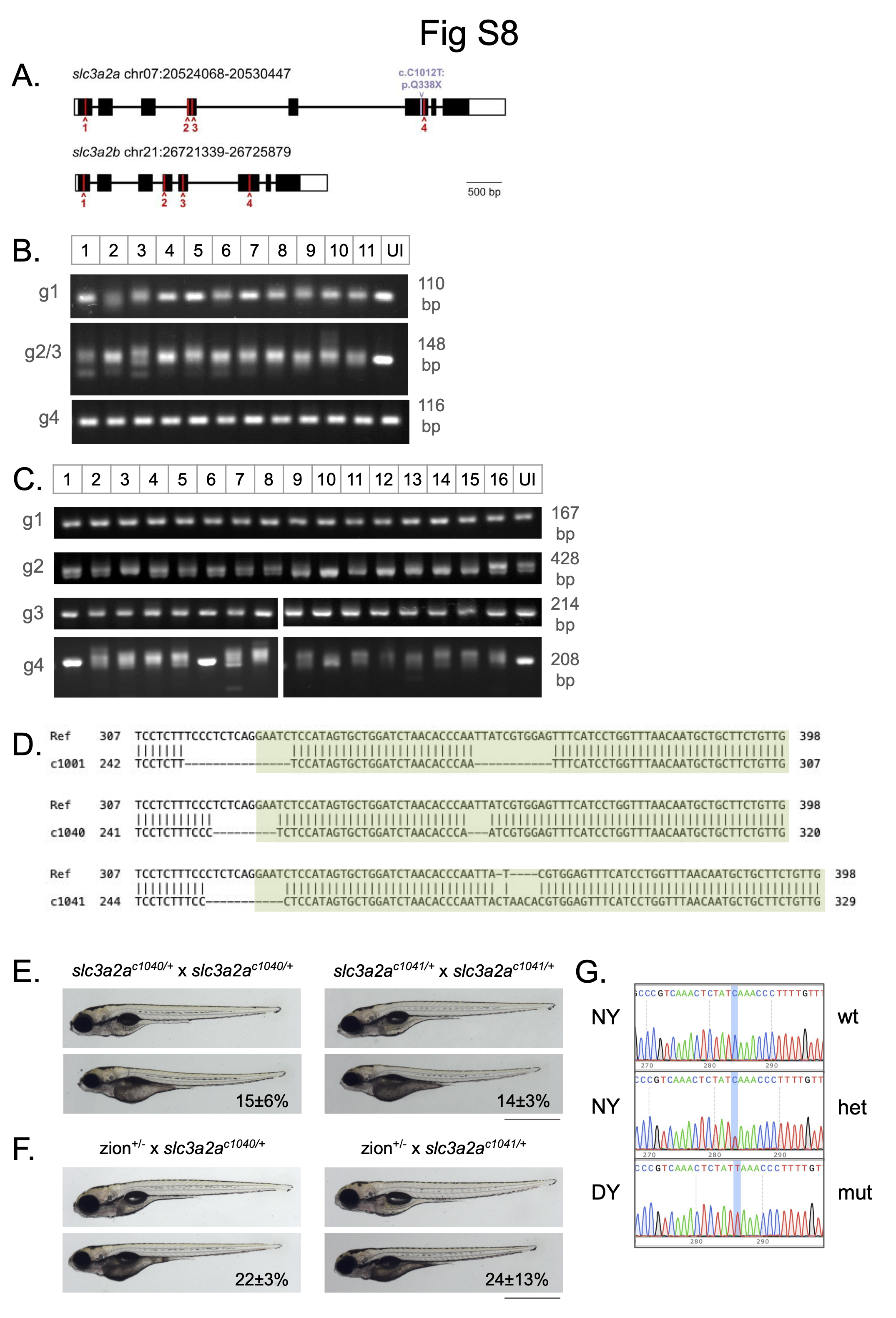

Supplement: S8 Fig — A) Schematic of the slc3a2a and slc3a2b genes. The location of the C > T base pair change in slc3a2a in the zion mutants is shown in purple. Red carats indicate locations targeted by CRISPR guides. B-C) PCR amplification around CRISPR guide sites in slc3a2a (B) and slc3a2b (C) injected animals. An uninjected animal (UI) was genotyped for comparison. “g#” indicates the guide site that is amplified for the respective gene, the expected size for each product is indicated on the right of each gel. For slc3a2a, editing is observed in many animals at g1 and g2/3; for slc3a2b, editing is observed in many animals at g4. D) c1001 (-15 and -11 bp), c1040 (-9 and -3 bp), and c1041 (-11 + 5 bp) slc3a2a alleles exhibit insertions/deletions in exon 4 and the exon 4 splice site as determined by Sanger Sequencing; exon 4 is highlighted in green. E) In-crossing slc3a2ac1040/+ (N = 2, n = 558) or slc3a2ac1041/+ (N = 2, n = 387) CRISPR mutants produces offspring with dark yolk. F) Outcrossing zion+/- to slc3a2ac1040/+ (N = 3, n = 299) or slc3a2ac1041/+ (N = 3, n = 219) produces offspring with dark yolk. For E-F, animals are 5 dpf, dark yolk frequency is reported as mean ± standard deviation, scale bar represents 1 mm. G) Sanger Sequencing of normal (NY) and dark yolk (DY) animals from a zion+/- in-cross have the expected genotype at the mutant position: wild-type (wt) - C, heterozygous (het) - C/T, mutant (mut) - T. (TIFF) [file pgen.1011702.s008.tiff]
