# Supplementary material for: Phenotype to genotype: A new and rapid approach using whole-genome sequencing
Source: PLoS Genet. 2025 Jul 14;21(7):e1011702. doi: 10.1371/journal.pgen.1011702 (PMC12273961; doi:10.1371/journal.pgen.1011702)
Supplement: S1 File — (DOCX) [file pgen.1011702.s009.docx]

**SUPPLEMENTARY MATERIALS AND METHODS**

**Ethics**

All animals were maintained, and procedures conducted, as detailed in Carnegie Institution’s Institutional Animal Care and Use Committee (IACUC) approved animal protocol #139.

**Fish Lines and husbandry**

Adult zebrafish (*Danio rerio*) were maintained at 27°C on a 14:10 h light:dark cycle and fed once daily with ~3.5% body weight Gemma Micro 300 (Skretting USA). Embryos were obtained by natural spawning and were raised in embryo medium at 28.5°C and kept on a 14:10 h light:dark cycle. On 3 dpf, plates were cleaned and fresh embryo medium was added. All lines including *slc45a2*^b4^, Fus(ApoBb.1-nanoluciferase) (Fus(ApoBb.1-nluc)), *mttp*^c655^, *mttp*^stl^ , *apobb.1^wz25^, mia2^mw91^*, and *dgat2*^sa13945^, were maintained in the AB background. Selected lines were outcrossed to WIK for mapping. All Zebrafish protocols were approved by the Carnegie Institution Department of Embryology Animal Care and Use Committee (Protocol #139).

**Brightfield Imaging**

3-5 dpf larvae were immobilized in cold 3% methylcellulose and imaged using a Nikon SMZ1500 microscope with HR Plan Apo 1x WD 54 objective, Infinity 3 Lumenera camera and Infinity Analyze 6.5 software.

**ENU mutagenesis**

ENU mutagenesis was performed as previously described[[50]](https://paperpile.com/c/Fz0DSR/MElWD) with some modifications. Specifically, 20 healthy and fecund AB male zebrafish were exposed to ENU (Millipore-Sigma, N3385) prepared at 3.3 mM in 10mM sodium phosphate buffer pH 6.5 in system water for 1 hour in a dark, quiet room. Tricaine (Millipore-Sigma, A5040) was added to a final concentration of 15 mg/L for the final 5 minutes of mutagenesis to minimize stress during mutagen removal. Mutagen was removed by draining the contaminated solution to a minimal volume (~100 mL) and diluting with wash solution (1.9L, 10 mg/mL tricaine in 10 mM phosphate buffered system water). After 3 washes, exposed fish were allowed to rest for 2 hours in wash solution before being transferred in a minimal volume (~100 mL) to an on-system 10 L tank with constant system water inflow and off-system drainage. After 48 and 96 hours, mutagen exposure was repeated for a total of 3 exposures. Mutagenized fish were rested 2 weeks before being bred to clear mutagenized sperm. All males survived initial ENU exposure, 17 survived past the 2 week resting period, and 13/17 remained fecund post-mutagenesis. ENU mutagenesis was performed in a dedicated space with appropriate personal protective equipment. All ENU contaminated solutions and materials were neutralized in a deactivation solution of 10% sodium thiosulfate 1% sodium hydroxide.

**Propagation and Screening**

Mutagenized (F0) males were crossed over two weeks to clear mosaic post-mitotic germ cells [[30]](https://paperpile.com/c/Fz0DSR/vaYgS) before outcrossing to *slc45a2*^b4^ to test for mutational load or to AB females to generate F1 fish. F1 fish were outcrossed to Fus(ApoBb.1-nluc)^+/-^ to generate F2 families. On 5 or 6 dpf, up to 50 larvae were put on the system to be raised for each F1. Once mature, F2 siblings were incrossed to generate F3 clutches for screening. Up to 100 larvae/clutch were collected and observed under a stereoscope using transmitted light at 3 and 5 dpf. On 5 dpf, larvae were anesthetized with Tricaine solution so that the yolks could be more easily assessed. An F2 family was considered fully screened once 6 unique clutches had been observed. F2 families that produced dark yolks at ~25% in one or more clutches were considered mutant families and were named and prioritized for characterization.

**Whole genome sequencing**

Larvae were pooled and flash frozen on dry ice then stored at -20°C. Genomic DNA was extracted using the DNeasy Blood & Tissue Kit (QIAGEN, 69504) according to the included protocol for Tissue. To achieve sufficient lysis, reagents were scaled 1.5X for pre-wash steps and the proteinase K digest was 15 minutes. DNA was eluted in nuclease-free water. DNA was prepared for sequencing using the Illumina DNA Prep, Tagmentation kit (Cat#20018705) and Nextera DNA CD Indexes (Cat#20018707) using an input of 200 ng and 5 PCR cycles per the user manual. Sequencing was performed on an Illumina NextSeq500 as a 150 bp single-end run with dual (8x8 bp indexing). ~30X coverage (~268 million reads) was acquired for each sample. Additional reads to reach 60X coverage were obtained on an Illumina NextSeq1000 as 200 bp single-end reads. The POLCA genome polishing software from the MaSuRCA genome assembly and analysis toolkit was used to align reads to the GRCz11 genome assembly and identify variance. Fastq files for published maize datasets were downloaded from the SRA database (SRR7467444, SRR7467445) and processed by POLCA as described above; ZM-b73 5.0 was used as the reference genome assembly.

**WheresWalker Output Analysis**

WheresWalker output files were analyzed and plotted in R (https://github.com/mfeltes/WheresWalkeR). The center of each gene was used as the coordinate for R plots. Centromere location was estimated according to previously reported markers (chr01: z1351, 33.9 Mb; chr17: z1408, 41.3 Mb, chr20: z3964, 16.7 Mb) [[51]](https://paperpile.com/c/Fz0DSR/3rWvt).

**PCR**

Genomic DNA for PCR was prepared by incubating whole larvae or adult fin clips in 50-100 µL 50 mM NaOH at 95°C for 15-20 min followed by neutralization with a 10% volume of 1 M Tris pH 8.0. Primers were designed using NCBI Primer Blast. PCR reactions were prepared using 4 µL Green GoTaq Flexi Buffer, 1.5 µL 25 mM MgCl_2_, 0.1 µL GoTaq Flexi DNA Polymerase (Promega, M8295), 0.5 µL dNTP Mix (Qiagen, 201901), 0.5 µL each of forward and reverse primers (10 µM, synthesized by Eurofins Scientific), and 1 µL of genomic DNA with nuclease-free water to 20 µL. PCR was performed for 35 cycles. Assay specific annealing temperatures, extension times, and oligo sequences are reported in supplementary file 2. PCR products were separated on a 2-3% agarose (Millipore Sigma, 11685678001) gel containing 0.5 µg/mL ethidium bromide (ThermoFisher, 15585001) and visualized with UV excitation.

Genotyping primers were designed using dCAPS finder 2.0[[52]](https://paperpile.com/c/Fz0DSR/5dWLc) to introduce a restriction enzyme cut site into either the wild-type or mutant PCR product. The *arches* allele was genotyped using primers SFMRF111 and SFMRF110^dCAPS^, the 205 bp product was digested with ApoI (NEB, R0566) and incubated for 1 h at 50°C to generate 205 bp (wild-type) and 179 bp (mutant) products. The *zion* allele was genotyped using primers SFMRF344^dCAPS^ and SFMRF346, the 116 bp product was digested with BccI (NEB, R0704) for 1 h at 37°C to generate 83 bp (wild-type) and 116 bp (mutant) products. Mttp alleles[[28]](https://paperpile.com/c/Fz0DSR/9OUkW) and ApoBb.1-nluc[[31]](https://paperpile.com/c/Fz0DSR/2nj9V) were genotyped as previously described. All primer/oligo sequences are reported in supplementary file 2.

**Recombination calculations**

Recombination frequency (Rf) was calculated as the fraction of animals with recombination out of the total number of animals observed multiplied by 100. Rf*cM was used to estimate the distance to the causative locus; a cM is 0.74 Mb in zebrafish[[24]](https://paperpile.com/c/Fz0DSR/hk66w). In some cases, for the *zion* mutants, genotype could not be determined by gel because the primers failed to adequately amplify the region, likely due to inefficient primer binding or amplification (Fig S7A-B). In some cases, unknown genotypes could be inferred based on the genotype of adjacent markers. Inferred genotypes were considered when calculating the average estimated position of the causative locus and are reported in the main text. Estimated positions calculated with only empirical genotypes are reported in Fig S7C.

**Sanger Sequencing**

PCR amplicons were prepared by standard PCR then sent to Genewiz for sanger sequencing. The *arches* amplicon was prepared with primers SFMRF111 and SFMRF104, and sequenced with SFMRF111. The *zion* amplicon was prepared with primers SFMRF460 and SFMRF346 and sequenced with SFMRF460. The *slc3a2a*(c1001) amplicon was prepared with primers SFMRF827 and SFMRF349 and sequenced with SFMRF347. All oligo sequences are reported in supplementary file 2.

**Lipoglo Assays**

ApoBb.1-nluc quantification and visualization was performed as previously described[[31]](https://paperpile.com/c/Fz0DSR/2nj9V). Briefly, for ApoBb.1-nluc quantification, animals were homogenized by sonication in a microplate-horn sonicator (Qsonica, Q700 sonicator with 431MPX microplate-horn assembly) in 100 µL stabilization buffer (1 g sucrose, 400 µL 0.5 M EGTA pH 8.0, 1 cOmplete, Mini, EDTA-free Protease Inhibitor Cocktail (Millipore Sigma, 11836170001), in 10 mL deionized water). 4-40 µL homogenate was diluted in reaction Buffer (0.5% Nano-Glo Luciferase Assay substrate, 25% Nano-Glo Luciferase Assay Buffer in PBS) to a final volume of 80 µL in an OptiPlate-96F Black flat-bottom plate. A SpectraMax M5 (Molecular Devices) or Tecan Spark plate reader set to top read with 500 ms (SpectraMax) or 20 ms (Tecan Spark) integration time was used to collect luminescence measurements. For ApoBb.1-nluc visualization in whole-mount animals, embryos were fixed in 4% paraformaldehyde (Electron Microscopy Science, 15714, diluted in PBS) for 3 h at room temperature then washed 3x15 minutes in 0.1% tween-20 (ThermoFisher Scientific, J66278.AP, diluted in PBS). Embryos were immobilized in 1% low melt agarose (Fisher Scientific, BP160, prepared in 1x TBE) containing 1% Nano-Glo Luciferase Assay substrate. Images were collected on a Zeiss Axiozoom V16 microscope V16 with a Zeiss AxioCam MRm. Luminescent signal was collected over 30 seconds with no illumination.

**Guide synthesis and F0 CRISPR**

Guide template oligo sequences were obtained from Wu et. al.[[19]](https://paperpile.com/c/Fz0DSR/AVcMz); template oligo and CRISPR tail primer sequences are reported in supplementary file 2. Oligos were synthesized by Eurofins Genomics. Guide RNA was prepared according to Wu et. al.[[19]](https://paperpile.com/c/Fz0DSR/AVcMz) with some modifications. Template DNA was prepared by PCR with CRISPR tail primer and Phusion High Fidelity Polymerase (ThermoFisher Scientific, F530). Template DNA was pooled according to target (*slc3a2a* = SFMRF324-327, *slc3a2b* = SFMRF404-407) then cleaned using QIAquick PCR Purification Kit (Qiagen, 28104). MEGAshortscript T7 Transcription Kit (ThermoFisher, AM1354) and 200-1000 ng template DNA was used to generate gRNA. gRNA was isolated by alcohol precipitation according to the kit protocol. The gRNA pellet was reconstituted in nuclease-free water, diluted to 2500 ng/µL, aliquoted, and stored at -80°C. An injection mix of 300 mM KCl, 0.1% phenol red, 0.8 µg/µL Alt-R S.p. Cas9 Nuclease V3 (IDT, 1081058) and 1000 ng/µL pooled gRNA in nuclease-free water was prepared. 2 nL of injection mix was injected into 1-cell stage zebrafish embryos. Larvae were raised in EM and observed on 3 and 5 dpf for yolk phenotypes.

***slc3a2a* line generation**

Guides targeting *slc3a2a* sites 2 and 3 (SFMRF325, SFMRF326) were synthesized and injected as described above except that guides were not pooled during synthesis and were combined at 500 ng/µL each in the final injection mix. Injected, F0 fish were raised to adulthood then in-crossed so that progeny could be screened for presence of the dark yolk phenotype. F0 pairs that produced dark yolk were outcrossed to AB and F1s were raised to adulthood. Genomic DNA was obtained from adult F1 fin clips to identify animals with editing at the *slc3a2a* locus by PCR amplifying a region that included guide cut sites 2 and 3 using primers SFMRF347 and SFMRF349. A male and female F1 with ~30 bp deletions were selected for further characterization. F1 fish were incrossed, generating F2 larvae with dark yolk. F1 and mutant F2 DNA was submitted for Sanger sequencing. F2 larvae were homozygous for a 26 bp deletion which removes part of exon 4, including the splice site acceptor. The same allele was detected in F1 parents which was detected using Poly Peak Parser[[53]](https://paperpile.com/c/Fz0DSR/Bnxwr). We named this allele *slc3a2a*^c1001^. F1 *slc3a2a*^c1001/+^ were crossed to *zion*^+/-^ to test for complementation. The same approach was used to generate, identify, and characterize alleles c1040 and c1041.

**Additional Software and Databases**

Google Sheets/Docs/Slides, Paperpile, SnapGene, Prism, and R were used to prepare materials for publication. Essential public databases include NCBI[[54]](https://paperpile.com/c/Fz0DSR/KH1ga), Ensembl[[55]](https://paperpile.com/c/Fz0DSR/sQJG5) and ZFIN[[56]](https://paperpile.com/c/Fz0DSR/AJXpZ).

**Data and Code Availability**

WGS datasets are available on the NCBI Sequence Read Archive (SRA) under BioProject PRJNA1187516. Accession numbers are provided in supplementary file 3. WheresWalker is publicly available on github at <https://github.com/alekseyzimin/WheresWalker>.
